# From Polyethylene Terephthalate Glycol (PETG) to 3D-Printed Resins: A Systematic Review and Meta-Analysis of Clear Aligner Material Performance and Safety

**DOI:** 10.7759/cureus.113427

**Published:** 2026-07-26

**Authors:** Fadi M Altawil, Roba I Abuzanoona

**Affiliations:** 1 Dentistry, Sinai University, Antwerpen, BEL

**Keywords:** 3d-printed resins, biocompatibility, clear aligners, mechanical properties, petg, tpu

## Abstract

Clear aligner therapy depends on polymeric materials that differ in stiffness, elasticity, force retention, surface stability, optical behavior, and biological safety. Polyethylene terephthalate glycol (PETG), thermoplastic polyurethane (TPU), polyurethane (PU), copolyesters, multilayer polymers, polypropylene/polyethylene (PP/PE)-based materials, and three-dimensional (3D)-printed resins are increasingly used, but their comparative performance remains heterogeneous. This systematic review and meta-analysis evaluated the mechanical performance, aging behavior, cytotoxicity, chemical release, microplastic release, and surface stability of orthodontic clear aligner materials. PubMed/MEDLINE, Scopus, Web of Science Core Collection, Cochrane Library, Google Scholar, Embase, and ScienceDirect were searched for English-language full-text studies published from January 2000 through June 23, 2026. Eligible studies evaluated material-specific mechanical, optical, biological, chemical, surface, or clinically relevant outcomes. Findings were synthesized narratively, and exploratory random-effects quantitative syntheses were conducted when numerical outcomes were sufficiently compatible. Methodological quality was evaluated using the Quality Assessment Tool for In Vitro Studies (QUIN) for the 22 laboratory investigations and the Joanna Briggs Institute (JBI) critical appraisal checklist for quasi-experimental studies for the prospective clinical/laboratory investigation. The review was not prospectively registered. Twenty-three studies were included, of which 22 were predominantly in vitro laboratory investigations. PETG, TPU, polyurethane, copolyesters, multilayer polymers, PP/PE-based materials, and direct 3D-printed resins showed substantial variation in modulus, flexural performance, stress relaxation, force decay, aging response, and surface roughness. QUIN assessment classified 18 in-vitro studies as having a medium risk of bias and four as having a high risk of bias; no study met the threshold for low risk of bias. Frequent methodological limitations included absent sample-size calculations, incomplete sampling descriptions, limited reporting of randomization and blinding, and inadequate reporting of operator or assessor calibration. Cytotoxicity findings were generally favorable under standard laboratory conditions, although reduced cell viability occurred with selected materials, concentrated extracts, or prolonged exposure. Evidence regarding BPA was limited, while other chemical leachables and microplastic release remained emerging concerns. Several quantitative outcomes showed high or extreme heterogeneity. Current evidence does not establish a universally superior clear aligner material. Material behavior and safety appear dependent on polymer composition, manufacturing method, thickness, post-processing, aging, and testing conditions. Because the evidence was predominantly laboratory-based, frequently at medium or high risk of bias, and methodologically heterogeneous, pooled estimates should not be translated directly into clinical material-selection or wear-schedule recommendations. Standardized laboratory methods and prospective clinical validation are required.

## Introduction and background

Clear aligner therapy has become an important alternative to fixed orthodontic appliances because it provides improved esthetics, removability, comfort, and easier oral hygiene control. Clear aligners are sequential, removable orthodontic appliances made from transparent polymeric materials and designed to move teeth gradually according to a digitally planned treatment sequence. Their increasing use reflects a major shift in orthodontic care, especially among patients who prefer less visible and more comfortable treatment options than conventional metal or ceramic brackets [1,2]. However, the success of clear aligner therapy depends not only on treatment planning and patient compliance but also on the ability of the aligner material to deliver accurate, sustained, and biologically safe orthodontic forces. Material stiffness, thickness, elasticity, stress relaxation, dimensional stability, aging resistance, transparency, and surface integrity directly influence force delivery, tooth movement, comfort, retention, and overall treatment efficiency [1,3]. Commonly studied aligner materials include polyethylene terephthalate glycol (PETG), thermoplastic polyurethane (TPU), polycarbonate, polypropylene, polyvinyl chloride (PVC) derivatives, multilayer polymers, and newer 3D-printable resins [1,4-6].

Clear aligners are fabricated from a diverse range of thermoplastic and resin-based materials, including PETG, TPU, PU, EVA, PP, PC, copolyesters, multilayer polymers, and direct 3D-printed resins. PETG is widely used because of its transparency and thermoformability, whereas TPU- and PU-based materials are generally more elastic. Multilayer systems attempt to combine stiffness and flexibility, while direct-printed resins may simplify fabrication and improve control of aligner geometry and thickness. However, the properties of commercial systems remain dependent on proprietary formulations, processing conditions, thermoforming, post-curing, and intraoral aging [1,4-6].

Mechanical properties and clinical force delivery

The orthodontic effectiveness of clear aligners depends on their ability to maintain force over time. Mechanical properties, such as elastic modulus, flexural strength, tensile strength, hardness, stress relaxation, fatigue resistance, creep resistance, and dimensional stability, determine how force is generated and transmitted to teeth. A material with inadequate stiffness may not provide sufficient force for complex movement, whereas excessive stiffness may reduce comfort, impair seating, or increase the risk of uncontrolled force delivery. Therefore, an ideal aligner material should provide enough force to move teeth while maintaining comfort, fit, and periodontal safety [1,3].

Thermoforming may reduce material performance by changing thickness, transparency, strength, surface roughness, and mechanical behavior. Studies summarized by Cenzato et al. [1] indicate that thermoforming can reduce thickness and force delivery in bending tests and may alter Young's modulus, tensile strength, and flexural properties. These changes are clinically important because aligner thickness is not uniform across all tooth surfaces after thermoforming, making force delivery difficult to predict. Intraoral aging further affects performance through saliva absorption, temperature fluctuations, pH changes, cyclic loading, masticatory stress, and cleaning procedures. Stress relaxation is especially relevant because aligners are worn for approximately 20-22 hours per day, often for 7, 10, or 14 days before replacement. Evidence suggests that many materials show rapid force decay during the first hours of use before reaching a plateau, which may reduce the efficiency of orthodontic movement if sustained forces are not maintained [1,4]. Complex movements, such as extrusion, rotation, torque, and bodily movement, may require materials with higher modulus, better fatigue resistance, and greater retention stability than simpler tipping movements.

Biocompatibility and cytotoxicity

Biocompatibility is a major concern because clear aligners remain in prolonged contact with saliva, gingiva, teeth, and oral mucosa. Cell viability is commonly used as the main outcome in cytotoxicity studies. International Organization for Standardization (ISO)-based thresholds often consider cell viability of 70% or higher as acceptable, while many orthodontic studies report values above 80% as favorable. Current evidence suggests that most clear aligner materials show acceptable biocompatibility, but some studies report mild or moderate cytotoxicity depending on material composition, extract concentration, exposure duration, thermoforming, resin curing, and post-processing methods [2,7-10].

Bandić et al. [7] evaluated four thermoplastic aligner materials before and after thermoforming in artificial saliva and found that some materials showed cell viability below the 70% threshold at higher concentrations and longer incubation times, although thermoforming itself did not significantly change cytotoxicity. Ferreira et al. [2] similarly concluded that most clear aligners appear generally safe, but some materials may show moderate cytotoxicity and mild inflammatory responses. For 3D-printed aligners, biocompatibility is closely linked to resin formulation, oxygen inhibition during curing, post-polymerization, extraction medium, and residual monomer release. Goracci et al. [6] reported that TC-85 generally showed no consistent cytotoxicity or estrogenic activity when properly processed, but also emphasized that evidence remains limited, heterogeneous, and mostly in vitro.

Bisphenol A (BPA) and chemical release

BPA and chemical release from clear aligners remain controversial. Some studies report no detectable BPA or only very low levels, while others suggest that aligners may release residual monomers, additives, or degradation products under specific conditions. This uncertainty is clinically relevant because patients wear aligners for many hours daily over months or years, leading to repeated exposure to any released compounds. BPA and other chemicals are commonly assessed in saliva, artificial saliva, water, or extraction media, but results vary according to analytical sensitivity, extraction time, material type, pH, temperature, mouthwash exposure, cleaning agents, and simulated aging protocols [2,6]. Goracci et al. [6] noted that TC-85 studies detected no BPA elution in some experiments, but variable urethane dimethacrylate release was reported under certain post-curing conditions. Therefore, although current evidence does not clearly prove clinically significant BPA exposure from aligners, long-term chemical safety remains insufficiently established.

Microplastic release and surface degradation

Recent evidence suggests that clear aligners may release microplastic particles during clinical wear or simulated loading. Microplastic release may result from surface roughening, abrasion, chewing forces, cyclic compression, parafunctional habits, brushing, cleaning procedures, and mechanical fatigue. De Stefano et al. [4] reported that clear aligners undergo surface changes after use, including roughness, microcracks, grooves, and abrasion. Their review found that microplastic particles can be released from PETG and TPU aligners, with commonly reported particle sizes around 5-20 μm. However, the evidence is still mostly laboratory-based or derived from early clinical aging studies. The systemic impact of aligner-derived microplastics remains uncertain, and standardized methods are needed to quantify particle release, characterize polymer type, and evaluate clinical relevance [4].

Clinical performance: Tooth movement, retention, comfort, and wear protocols

The clinical relevance of an aligner material depends on the interaction of stiffness, elasticity, thickness, stress relaxation, fit, and resistance to intraoral aging. Laboratory findings suggest that these properties can affect force delivery and retention; however, direct evidence linking specific materials with tooth-movement accuracy, patient comfort, or optimal replacement intervals remains limited. Differences among 7-day, 10-day, and 14-day wear protocols, as well as the effects of cleaning methods, beverages, heat, and patient handling, therefore require clinical rather than solely laboratory validation [1-4].

Research gap

Although several reviews have summarized clear aligner materials, important gaps remain. First, mechanical testing methods are not standardized across studies, making direct comparison between PETG, TPU, multilayer materials, EVA, PP, PC, and 3D-printed resins difficult. Second, limited evidence directly connects material properties with specific orthodontic movements, such as intrusion, extrusion, tipping, rotation, and bodily movement. Third, wear protocols are not consistently evaluated, especially regarding whether 7-day, 10-day, or 14-day wear affects force decay differently across materials. Fourth, the effects of cleaning methods, mouthwash exposure, hot water, pH variation, and patient maintenance habits remain underexplored. Fifth, evidence on BPA release, chemical leaching, and microplastic dispersion is inconsistent and often based on laboratory models rather than long-term clinical data. Finally, direct comparisons between thermoformed materials and 3D-printed aligner resins remain limited, particularly in clinical settings, where patient compliance, oral environment, and functional loading may substantially alter material behavior [4-6]. Unlike previous reviews that have focused primarily on mechanical properties, biological safety, BPA release, microplastic dispersion, or direct-printing technologies as separate topics, the present review integrates mechanical performance, thermoforming and aging behavior, cytotoxicity, chemical leaching, microplastic release, surface degradation, and clinically relevant laboratory outcomes across both conventional thermoformed and direct 3D-printed aligner materials. It also provides an exploratory quantitative synthesis of compatible outcomes and evaluates the methodological quality of the predominantly in-vitro evidence using the Quality Assessment Tool for In Vitro Studies (QUIN) tool. This integrated approach represents the principal novelty of the review.

Aim of the review

This systematic review aims to evaluate the mechanical properties, biocompatibility, chemical safety, microplastic release, and clinical relevance of different clear aligner materials.

Research questions

The following are the research questions: Which clear aligner materials show the best mechanical stability after thermoforming and aging? How do PETG, TPU, multilayer polymers, EVA, PP, PC, and 3D-printed resins compare in force retention and stress relaxation? What is the biocompatibility profile of different aligner materials? Is BPA or chemical release clinically significant in clear aligner materials? Do clear aligners release microplastics during wear or simulated loading? Which material properties are associated with better clinical performance and retention? Are 3D-printed aligner resins comparable or superior to thermoformed materials?

## Review

Methodology

Study Design

This study was designed as a systematic review and meta-analysis evaluating the material performance and safety of orthodontic clear aligner materials. The review focused on thermoplastic and resin-based materials used for clear aligner fabrication, including PETG, thermoplastic polyurethane (TPU), polyurethane (PU), ethylene-vinyl acetate (EVA), polypropylene (PP), polycarbonate (PC), polypropylene/polyethylene (PP/PE)-based materials, polyvinyl chloride (PVC) derivatives, copolyesters, multilayer polymers, and three-dimensional (3D)-printed aligner resins. The review was conducted according to the Preferred Reporting Items for Systematic Reviews and Meta-Analyses (PRISMA) framework. The primary outcomes were mechanical performance, including elastic modulus, flexural strength, tensile strength, stress relaxation, force decay, hardness, surface roughness, and thickness change. Secondary outcomes included transparency or optical change, cell viability, cytotoxicity, BPA release, chemical leaching, microplastic release, retention force, tooth movement accuracy, and patient comfort. This systematic review was not prospectively registered, and no publicly accessible protocol was prepared. The eligibility criteria, outcome domains, data-extraction framework, and synthesis approach described in this manuscript represent the procedures used for the completed review. The absence of prospective registration limits the ability to compare the completed review with a time-stamped prespecified protocol and is therefore acknowledged as a methodological limitation.

Databases and Search Strategy

A comprehensive electronic search was conducted in PubMed/MEDLINE, Scopus, Web of Science Core Collection, Cochrane Library, Google Scholar, Embase, and ScienceDirect for studies published from January 2000 through June 23, 2026. The database-specific search strings, Boolean operators, search fields, filters, and execution date are presented in Table 1.

**Table 1:** Search strategy used for study identification

Database	Search terms or search string	Filters or limits	Search date	Purpose
PubMed	("clear aligner" OR "orthodontic aligner") AND ("polyethylene terephthalate glycol" OR PETG OR "thermoplastic polyurethane" OR TPU OR polyurethane OR thermoplastic OR "3D printed resin" OR "three-dimensional printed resin") AND ("mechanical properties" OR "elastic modulus" OR "flexural strength" OR "stress relaxation" OR "force decay" OR aging OR thermoforming OR biocompatibility OR cytotoxicity OR "bisphenol A" OR BPA OR "chemical release" OR microplastic)	English; full text; 2000 onward	June 23, 2026	To identify biomedical and dental studies on clear aligner materials and their performance or safety outcomes.
Scopus	TITLE-ABS-KEY (“clear aligner” OR “orthodontic aligner”) AND TITLE-ABS-KEY (PETG OR TPU OR polyurethane OR thermoplastic OR “3D printed resin” OR “TC-85”) AND TITLE-ABS-KEY (“mechanical properties” OR “force decay” OR “stress relaxation” OR cytotoxicity OR “chemical leaching” OR microplastic)	English; article; 2000 onward	June 23, 2026	To capture multidisciplinary materials science and dental literature.
Web of Science	TS = (“clear aligner” OR “orthodontic aligner”) AND TS = (PETG OR TPU OR polyurethane OR thermoplastic OR “3D printed resin” OR “TC-85”) AND TS = (“elastic modulus” OR “flexural strength” OR “surface roughness” OR cytotoxicity OR “BPA release” OR microplastic)	English; article; 2000 onward	June 23, 2026	To identify indexed material, performance, and safety studies.
Cochrane Library	("clear aligner" OR "orthodontic aligner") AND (material OR thermoplastic OR polyurethane OR cytotoxicity OR biocompatibility)	English; 2000 onward	June 23, 2026	To identify clinical or controlled evidence where available.
Google Scholar	“clear aligner PETG TPU mechanical properties thermoforming aging”; “clear aligner biocompatibility cytotoxicity BPA release”; “clear aligner microplastic release”; “3D printed clear aligner resin TC-85 mechanical properties”	Studies published before January 2000 were excluded.	June 23, 2026	To identify additional grey or cross-disciplinary literature not captured in database searches.
Embase	("clear aligner" OR "orthodontic aligner") AND (PETG OR TPU OR polyurethane OR "3D printed resin") AND (cytotoxicity OR biocompatibility OR "mechanical properties")	English; full text; 2000 onward	June 23, 2026	To identify biomedical and pharmacological safety-related studies.
ScienceDirect	"clear aligner material" AND (thermoplastic OR polyurethane OR PETG OR microplastic OR cytotoxicity OR "force decay")	English; 2000 onward	June 23, 2026	To identify materials science studies and full-text experimental reports.
Manual reference screening	Reference lists of included articles and relevant reviews were screened manually.	Relevant original studies only	June 23, 2026	To identify additional eligible studies missed by electronic searches.

Reference lists of eligible articles and relevant reviews were also examined manually to identify potentially eligible primary studies. For Google Scholar, the first 100 results returned for each prespecified query were screened in order of relevance. The search was designed to identify studies evaluating orthodontic clear aligner materials, including thermoplastic and 3D-printed resin systems, and their mechanical, biological, chemical, optical, and surface-degradation outcomes.

Eligibility Criteria

Studies were selected according to predefined inclusion and exclusion criteria (Table 2).

**Table 2:** Inclusion and exclusion criteria

Domain	Inclusion criterion	Exclusion criterion
Topic relevance	Studies evaluating orthodontic clear aligner materials.	Studies not related to orthodontic clear aligners.
Material type	Studies involving polyethylene terephthalate glycol (PETG), thermoplastic polyurethane (TPU), polyurethane (PU), ethylene-vinyl acetate (EVA), polypropylene (PP), polycarbonate (PC), polypropylene/polyethylene (PP/PE)-based materials, polyvinyl chloride (PVC) derivatives, copolyesters, multilayer polymers, or three-dimensional (3D)-printed aligner resins.	Studies without material-specific data.
Appliance type	Studies evaluating materials used for clear aligners.	Studies evaluating only fixed appliances or brackets.
Retainers	Retainer studies were eligible only when the tested material was also applicable to clear aligners.	Retainer-only studies were excluded when the material was not clearly applicable to clear aligners.
Study design	In vitro, ex vivo, in vivo, clinical, observational, comparative, and experimental studies were eligible.	Narrative reviews, editorials, letters, conference abstracts, and opinion articles were excluded from quantitative synthesis.
Mechanical outcomes	Studies reporting elastic modulus, flexural strength, tensile strength, stress relaxation, force decay, hardness, surface roughness, thickness change, retention force, or tooth movement accuracy were eligible.	Studies without extractable mechanical outcome data were excluded.
Optical outcomes	Studies reporting transparency, light transmittance, color stability, or other optical change were eligible.	Studies without relevant optical or material-performance outcomes were excluded.
Biological outcomes	Studies reporting cell viability, cytotoxicity, biocompatibility, or patient comfort were eligible.	Animal studies were excluded unless directly relevant to material biocompatibility.
Chemical outcomes	Studies reporting bisphenol A (BPA) release, bisphenol S (BPS) release, monomer release, or chemical leaching were eligible.	Studies without extractable chemical or safety data were excluded.
Microplastic outcomes	Studies reporting microplastic release, nanoplastic release, particle count, particle size, or particle morphology were eligible.	Studies discussing microplastics without original extractable data were excluded from quantitative synthesis.
Language	English-language studies were included.	Non-English articles were excluded.
Publication period	Full-text articles published from 2000 onward were included.	First 100 results for each prespecified query, ranked by relevance; English-language records; 2000 to June 23, 2026
Full text	Studies with full-text availability were included.	Studies with unavailable full text were excluded.
Duplicates	The most complete version of a study was retained.	Duplicate publications were excluded.
Reporting clarity	Studies with clear testing protocols and extractable outcomes were included.	Studies with unclear testing protocols or missing outcome data were excluded.

PICO (Population, Intervention/Exposure, Comparator, and Outcome) Framework and Study Selection

The population was defined as clear aligner materials used in orthodontic treatment. The intervention or exposure consisted of different aligner materials, including PETG, TPU, multilayer polymers, EVA, PP, PC, and 3D-printed resins. Comparators included other aligner materials, pre- versus post-thermoforming conditions, pre- versus post-aging conditions, different immersion or loading environments, and control materials. Outcomes included mechanical properties, biocompatibility, BPA or chemical release, microplastic release, surface degradation, and clinical performance.

All identified records were imported into EndNote 21 (Clarivate, Philadelphia, PA). Duplicate detection was performed electronically and was supplemented by manual comparison of titles, authors, publication years, and digital object identifiers. No duplicate records were identified during this process. Two reviewers independently screened titles and abstracts against the predefined eligibility criteria, followed by full-text assessment of potentially eligible reports. Disagreements were resolved through discussion, and a third reviewer was consulted when consensus could not be reached. A total of 620 records underwent title and abstract screening, of which 560 were excluded. Sixty full-text reports were retrieved and assessed for eligibility. Thirty-seven reports were excluded because they were unrelated to orthodontic clear aligner materials, lacked extractable material-specific data, represented reviews or editorials, focused exclusively on retainers or fixed appliances, or had unavailable or insufficiently reported outcomes. Twenty-three studies were included in the qualitative synthesis, and sufficiently compatible numerical comparisons were considered for the quantitative synthesis.

Data Extraction

Two reviewers independently extracted data using a standardized extraction form. Extracted variables included author, year, country, study design, material type, brand or product, sample size, comparator, manufacturing method, thermoforming status, aging medium, aging duration, temperature, cyclic loading, outcome measures, numerical values, standard deviations, confidence intervals, and main findings. Separate extraction tables were prepared for general study characteristics, material properties, mechanical testing characteristics, cytotoxicity outcomes, BPA and chemical release, microplastic release and surface roughness, and clinical performance outcomes. Disagreements between reviewers were resolved through discussion, and a third reviewer was consulted where necessary.

Methodological Quality and Risk-of-Bias Assessment

Methodological quality was assessed using appraisal tools selected according to study design. The 22 in vitro laboratory investigations were assessed using the QUIN, which was developed and validated specifically for dental laboratory research. The prospective clinical/laboratory study by Divakar et al. was assessed separately using the Joanna Briggs Institute (JBI) critical appraisal checklist for quasi-experimental studies [10].

The QUIN tool evaluates 12 criteria: clearly stated aims or objectives, sample-size calculation, sampling technique, comparison-group details, methodological description, operator details, randomization, outcome-measurement method, outcome-assessor details, blinding, statistical analysis, and presentation of results. Each applicable criterion was classified as adequately specified and assigned 2 points, inadequately specified and assigned 1 point, or not specified and assigned 0 points. Criteria that were genuinely not applicable were excluded from the denominator. The final percentage was calculated using the following formula: total score × 100/(2 × number of applicable criteria). Scores above 70% were classified as low risk of bias, scores from 50% to 70% as medium risk of bias, and scores below 50% as high risk of bias. Two reviewers independently completed the methodological appraisals. Disagreements were resolved through discussion and consensus. The appraisal findings were used to inform the interpretation of the evidence but were not used to exclude studies or alter their statistical weights. Complete study-level QUIN judgments are presented in Table 3.

**Table 3:** QUIN methodological quality assessment of the included in vitro studies C1, clearly stated aims/objectives; C2, sample-size calculation; C3, sampling technique; C4, comparison group; C5, methodology; C6, operator details; C7, randomization; C8, outcome measurement; C9, outcome-assessor details; C10, blinding; C11, statistical analysis; C12, presentation of results; QUIN, Quality Assessment Tool for In Vitro Studies Scores were assigned as adequately specified = 2, inadequately specified = 1, and not specified = 0. Final scores above 70% indicated low risk of bias, scores of 50%-70% indicated medium risk, and scores below 50% indicated high risk.

Study	Study design	C1 Aim	C2 Sample Size	C3 Sampling	C4 Comparator	C5 Methodology	C6 Operator	C7 Randomization	C8 Outcome Measurement	C9 Outcome Assessor	C10 Blinding	C11 Statistics	C12 Results	Total Score	Applicable Criteria	Final Score	Risk of Bias
Kwok et al. (2022) [8]	In vitro experimental material characterization	2	0	1	2	2	0	0	2	0	0	1	2	12	12	50.00%	Medium
Xiang et al. (2021) [9]	In vitro simulated oral-environment study	2	0	1	2	1	0	0	2	0	0	1	2	11	12	45.80%	High
Dalaie et al. (2021) [11]	In vitro experimental study	2	0	1	2	2	0	0	2	0	0	1	2	12	12	50.00%	Medium
Cui et al. (2025) [12]	In vitro experimental study	2	0	1	2	2	0	0	2	0	0	1	2	12	12	50.00%	Medium
Elshazly et al. (2023) [13]	In vitro biomechanical study	2	0	1	2	2	0	0	2	0	0	1	2	12	12	50.00%	Medium
Fang et al. (2013) [14]	In vitro experimental study	2	0	1	2	2	0	0	2	0	0	1	2	12	12	50.00%	Medium
Bhate and Nagesh (2024) [15]	In vitro experimental study	2	0	1	2	2	0	0	2	0	0	1	2	12	12	50.00%	Medium
Das et al. (2026) [16]	Prospective double-blind in vitro cytotoxicity study	2	0	1	2	2	0	0	2	0	1	1	2	13	12	54.20%	Medium
Pendse et al. (2025) [17]	In vitro cytotoxicity study	2	0	1	2	1	0	0	1	0	0	1	1	9	12	37.50%	High
Zecca et al. (2025) [18]	In vitro comparative microscopic study	2	0	1	2	2	0	0	2	0	0	1	2	12	12	50.00%	Medium
Puchert et al. (2026) [19]	In vitro experimental material study	2	0	1	2	2	0	0	2	0	0	1	2	12	12	50.00%	Medium
Šimunović et al. (2023) [20]	In vitro comparative material-aging study	2	0	1	2	2	0	0	2	0	0	1	2	12	12	50.00%	Medium
Chen et al. (2025) [21]	In vitro direct-printed vs thermoformed material study	2	1	1	2	2	0	0	2	0	0	1	2	13	12	54.20%	Medium
Cintora-López et al. (2023) [22]	In vitro experimental study	2	0	1	2	2	0	0	2	0	0	1	2	12	12	50.00%	Medium
Chen et al. (2023) [23]	In vitro force-decay study	2	0	1	2	2	0	0	2	0	0	1	2	12	12	50.00%	Medium
Daniele et al. (2020) [24]	In vitro physicochemical/mechanical characterization	2	0	1	2	2	0	0	2	0	0	1	2	12	12	50.00%	Medium
Lo et al. (2024) [25]	In vitro cytotoxicity study	2	0	1	2	2	0	0	2	0	0	1	1	11	12	45.80%	High
Bandić et al. (2025) [7]	In vitro cytotoxicity study	2	0	1	2	2	0	0	2	0	0	1	1	11	12	45.80%	High
Wendl et al. (2026) [26]	In vitro analytical leaching/biocompatibility study	2	0	1	2	2	0	0	2	0	0	1	2	12	12	50.00%	Medium
Orilisi et al. (2026) [27]	In vitro multidisciplinary microplastic-release study	2	0	1	2	2	0	0	2	0	0	1	2	12	12	50.00%	Medium
Choi et al. (2025) [28]	In vitro mechanical/viscoelastic study	2	0	1	2	2	0	0	2	0	0	1	2	12	12	50.00%	Medium
Khalil and Zaher (2025) [29]	In vitro comparative flexural-strength study	2	0	1	2	2	0	0	2	0	0	1	2	12	12	50.00%	Medium

Data Synthesis and Meta-Analysis

A narrative synthesis was conducted for all included studies and organized according to material category, manufacturing method, experimental condition, and outcome domain. Quantitative synthesis was undertaken when outcomes were reported on a common scale or could be transformed into a compatible effect measure.

Statistical analyses were performed using Stata/MP (version 18.0; StataCorp LLC, College Station, TX). Random-effects models estimated using restricted maximum likelihood were selected because substantial heterogeneity was expected in polymer composition, specimen geometry, material thickness, manufacturing or thermoforming procedures, aging media, temperature, loading conditions, exposure duration, and outcome measurement. Comparative continuous outcomes were summarized using mean differences or Hedges' g. Single-group continuous outcomes were summarized using pooled means, while cytotoxicity classifications were summarized using logit-transformed proportions. All estimates were reported with 95% confidence intervals, and statistical significance was defined using a two-sided P value below 0.05. Statistical heterogeneity was assessed using the I² statistic.

Several datasets contained multiple material groups, exposure conditions, or testing configurations derived from the same source study. These observations may be statistically correlated and were not considered fully independent replications. Quantitative findings were therefore treated as exploratory. Syntheses containing data from at least two independent source studies were described as meta-analyses, whereas calculations based on multiple experimental conditions from a single source study were described as within-study quantitative summaries.

Formal meta-regression, funnel-plot asymmetry testing, and statistical publication-bias tests were not undertaken because fewer than 10 independent studies contributed to each outcome. Where heterogeneity was high or extreme, the summary estimate was interpreted as a descriptive average across heterogeneous laboratory conditions rather than evidence of a common material effect.

Results

A total of 23 studies were included in this systematic review, covering a broad range of orthodontic clear aligner materials, including PETG, TPU, PU, copolyester, multilayer polymers, PP/PE-based materials, and direct 3D-printed resins. Most included studies were in vitro experimental investigations, while a smaller number involved simulated aging, intraoral wear, cytotoxicity testing, chemical leaching, or microplastic-release assessment. The reported outcomes included mechanical performance, stress relaxation, force decay, surface roughness, flexural strength, elastic modulus, biocompatibility, BPA or chemical release, microplastic dispersion, and indirect indicators of clinical performance.

**Figure 1:**
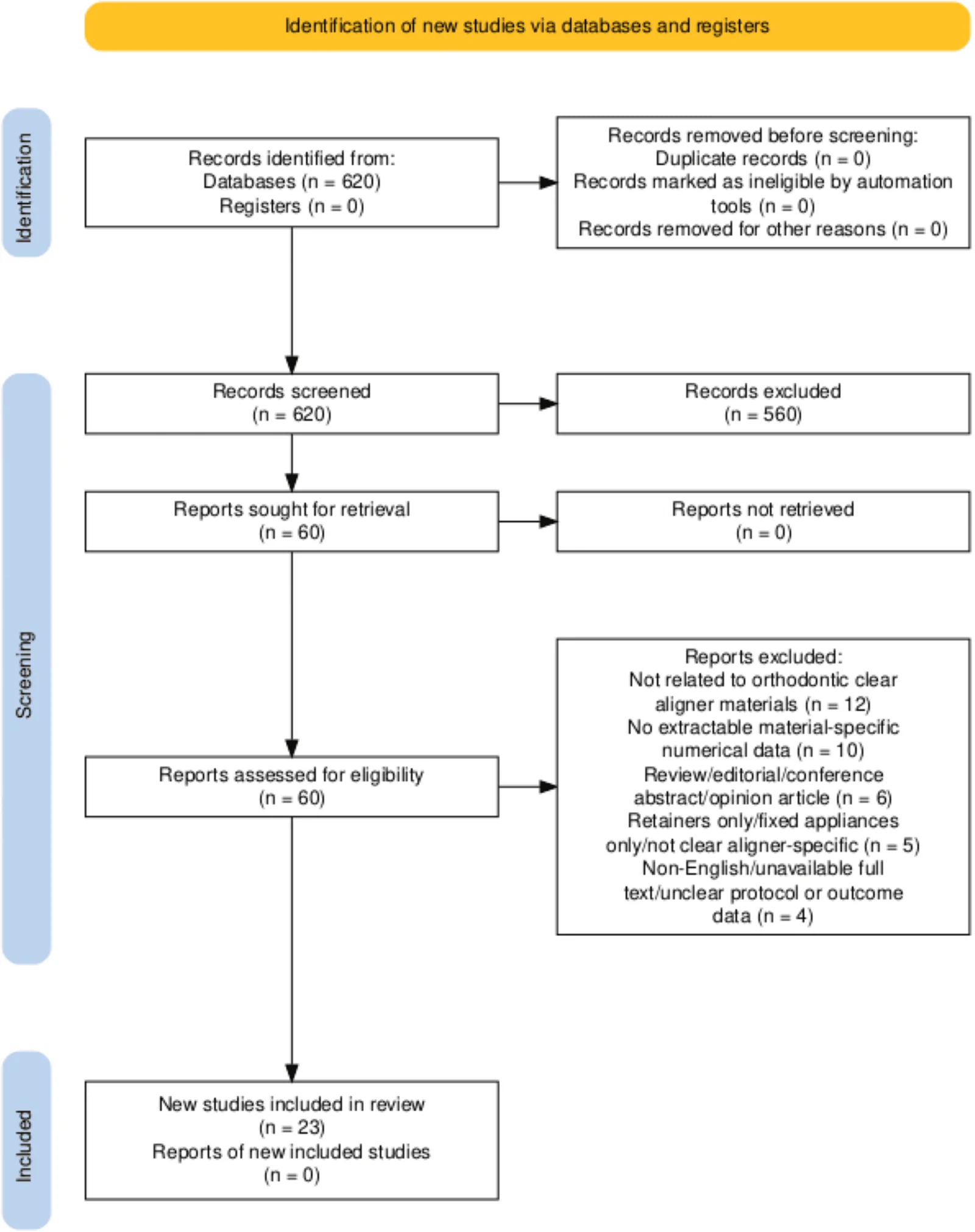
PRISMA flow chart PRISMA: Preferred Reporting Items for Systematic Reviews and Meta-Analyses

The PRISMA flow diagram shows that 620 records were identified through database searching, with no additional records from registers. No duplicates or automatically excluded records were removed before screening. After title and abstract screening, 560 records were excluded, leaving 60 reports for retrieval and eligibility assessment. All 60 reports were successfully retrieved and assessed in full text. Of these, 37 reports were excluded for reasons including lack of relevance to orthodontic clear aligner materials, absence of extractable material-specific numerical data, review or editorial design, retainer-only or non-aligner-specific focus, and unavailable or unclear full-text/outcome data. Finally, 23 studies met the eligibility criteria and were included in the systematic review (Figure 1).

Twenty-two in vitro studies were evaluated using the QUIN tool (Table 3). Eighteen studies were classified as having a medium risk of bias, while four studies, Xiang et al., Pendse et al., Lo et al., and Bandić et al. [7,9,17,25], were classified as having a high risk of bias. No in vitro study met the threshold for low risk of bias. Across the evidence base, study aims, comparison groups, experimental procedures, outcome-measurement methods, and principal results were generally described adequately. However, a priori sample-size calculations, specimen-selection procedures, operator training or calibration, randomization, outcome-assessor details, blinding, and complete statistical reporting were frequently absent or insufficiently described. Divakar et al. was appraised separately because it included a prospective clinical intraoral-wear component [10]. The JBI appraisal of Divakar et al. indicated that the temporal sequence between intraoral exposure and outcome measurement was clear, repeated measurements were obtained at predefined time points, the outcomes were measured consistently, the measurement methods were considered reliable, and the statistical approach was generally appropriate. The absence of a separate control group was a methodological limitation, follow-up completeness was insufficiently described, and criteria concerning comparability between separate participant groups and equal co-interventions were considered not applicable because the study used a single prospective cohort. Overall, the study provided useful clinical aging data but remained susceptible to bias arising from its small sample, lack of random allocation, and limited control of patient-level intraoral exposures.

Table 4 summarizes the general characteristics of the 23 included studies, showing that most evidence was derived from in vitro experimental designs, with fewer studies involving clinical intraoral aging, cytotoxicity testing, leaching analysis, or simulated microplastic release. The included studies covered a broad range of clear aligner materials, including PETG, TPU, PU, copolyester, PP/PE-based materials, multilayer polymers, and direct 3D-printed resins. The table highlights the methodological diversity of the evidence base and shows that material performance was mainly assessed through mechanical, biological, chemical, optical, and surface-degradation outcomes [7-29] (Table 4).

**Table 4:** General characteristics of the included studies AFM, atomic force microscopy; BPA, bisphenol A; BPS, bisphenol S; DMA, dynamic mechanical analysis; DPA, direct printed aligner; DSC, differential scanning calorimetry; FTIR, Fourier-transform infrared spectroscopy; GPa, gigapascal; MP, microplastic; PET, polyethylene terephthalate; PETG/PET-G, polyethylene terephthalate glycol; PCT, polycyclohexylenedimethylene terephthalate; PMMA, polymethyl methacrylate; PP/PE, polypropylene/polyethylene; PP-EPR, polypropylene-ethylene-propylene rubber; PU, polyurethane; SEM, scanning electron microscopy; SMP, shape-memory polymer; T0, baseline/as received; T1, post-thermoforming; T2, post-aging or post-intraoral use; TFA, thermoformed aligner; TPU, thermoplastic polyurethane

Study	Country	Design	Material/Brand	Sample/Comparator	Main Outcome and Key Finding
Kwok et al. (2022) [8]	USA	In vitro material characterization	TPU, PETG/PCT, PP-EPR, PCT/TPU; SmartTrack, Zendura, Essix Ace, Essix C+, Tru-Tain DX30-25	n = 5/material; five commercial materials compared	Chemical, thermal, transparency, and flexural properties were evaluated. Flexural modulus ranged from 0.9-1.7 GPa, with Zendura showing the highest stiffness.
Xiang et al. (2021) [9]	China	In vitro simulated oral study	Conventional and modified PETG aligners	90 aligners; PETG types compared across activation levels and immersion periods	Force stability and surface morphology were assessed. Modified PETG showed slower force decay and better surface stability after artificial saliva aging.
Divakar et al. (2026) [10]	India	Prospective clinical/laboratory study	PETG and PU; Erkodur, Taglus, Zendura	15 patients; T0, T1, and T2 compared	Thermoforming and 2-week intraoral aging altered roughness, hardness, and flexural strength. PETG showed increased roughness, while PU showed reduced flexural strength.
Dalaie et al. (2021) [11]	Iran	In vitro experimental study	PETG; Duran, Erkodur	96 samples; untreated, thermoformed, and aged sheets compared	Thermoforming reduced thermal and mechanical properties, while simulated aging had a limited additional effect. Duran was more stable than Erkodur.
Cui et al. (2025) [12]	China	In vitro experimental study	PETG, copolyester, PP/PE; Biolon, Duran, Erkodur, Essix ACE, Essix C+	Tensile and relaxation specimens tested under temperature/wet conditions	Elevated temperature accelerated stress relaxation. Erkodur and Essix ACE showed relatively stable behavior.
Elshazly et al. (2023) [13]	Germany	In vitro biomechanical study	Copolyester/PET-based material; Essix ACE	10 thermoformed aligners; water vs artificial saliva	Aging medium had no significant effect on force or torque decay over 14 days.
Fang et al. (2013) [14]	China	In vitro experimental study	PETG, PET, copolyester; Erkodur, Biolon, Masel, Keystone, Duran	30 specimens; air vs 37°C water bath	Residual stress declined over time and faster in water. Erkodur and Masel showed slower relaxation.
Bhate and Nagesh (2024) [15]	India	In vitro experimental study	PET-G and PU; Duran, Zendura	24 samples; T0, T1, and T2 compared	Thermoforming and aging reduced flexural strength. Zendura showed increased surface roughness after aging.
Das et al. (2026) [16]	India	In vitro cytotoxicity study	Commercial aligners: Invisalign, Smile, Illusion	One aligner set/system; eluents compared with control	Invisalign showed the highest biocompatibility, Smile was acceptable, and Illusion showed dose-dependent cytotoxicity.
Pendse et al. (2025) [17]	India	In vitro cytotoxicity study	PET-G, PET, multilayer PU; TAC, Flash, Taglus Premium, Invisalign SmartTrack	Four materials were tested in triplicate.	No statistically significant cytotoxicity was observed among the tested materials.
Zecca et al. (2025) [18]	Italy	In vitro microscopic analysis	3D-printed resin and thermoformed aligner; Graphy TC-85-DAC, Invisalign SmartTrack	DPA vs TFA samples	DPA released larger and more numerous plastic particles, while TFA showed higher AFM surface roughness.
Puchert et al. (2026) [19]	Germany	In vitro material study	3D-printed materials and PET-G; Dental LT Clear V2, V Print Splint Comfort, TC-85 DAC, Biolon	Multiple specimen groups; dry vs water-stored conditions	Water storage reduced the modulus and hardness of 3D-printed materials, while Biolon PET-G remained more stable.
Šimunović et al. (2023) [20]	Croatia	In vitro material-aging study	PETG, TPU, multilayer materials; Duran+, CA Pro, Zendura A, Zendura FLX	96 samples; control vs 500/1000 thermocycles	Duran+ showed significant modulus change after 1000 thermocycles; roughness and FTIR findings remained largely stable.
Chen et al. (2025) [21]	Taiwan	In vitro comparative study	3D-printed and thermoformed materials; LC, GY, RD, ED, Biolon SC	n = 12/material/outcome	Graphy GY showed the best overall 3D-printed performance and was comparable to thermoformed materials.
Cintora-López et al. (2023) [22]	Spain	In vitro experimental study	Polyurethane clear aligner material	60 aligners; saliva vs thermocycling and different loads	Thermal cycling and higher force reduced the constant deformation phase; water absorption remained below 0.4%.
Chen et al. (2023) [23]	Taiwan	In vitro experimental study	PETG, PET, TPU/PU; Duran T, Biolon, Zendura FLX	360 samples; saliva and beverages compared	All materials showed force decay over time. Zendura FLX showed better resistance to beverage-related strength loss.
Daniele et al. (2020) [24]	Italy	In vitro physicochemical/mechanical study	PETG/PET and PU; Erkodur, Essix, Ghost Aligner, Zendura	Mechanical, optical, and water absorption tests	Zendura had the highest modulus and tensile strength, but lower transparency and higher water absorption.
Lo et al. (2024) [25]	Taiwan	In vitro cytotoxicity study	PETG, TPU, PET; Duran, Essix, Leone, Maxflex, Zendura, Keystone	Triplicate experiments; original vs thermoformed samples	Most materials maintained cell viability above 70%; thermoformed PETG showed reduced viability after 14-day extraction.
Bandić et al. (2025) [7]	Croatia	In vitro cytotoxicity study	TPU and multilayer materials; Zendura A, Zendura FLX, Erkoloc-pro, CA Pro+	Triplicate testing; non-thermoformed vs thermoformed	Cytotoxicity occurred only in selected high-concentration/long-exposure conditions; thermoforming had no major effect.
Wendl et al. (2026) [26]	Germany/Austria	In vitro leaching/biocompatibility study	Thermoformed, 3D-printed, and SMP aligners; Graphy, SMP-Aligner, CA Pro, Erkodur-al	Four materials: leachable and bisphenol testing	BPA was not detected; BPS was detected only in Graphy. Graphy and SMP released more leachable compounds.
Orilisi et al. (2026) [27]	Italy	In vitro microplastic-release study	PET/PETG, PU, PMMA-based resin, mixed materials; 10 brands	n = 3 pairs/brand/time point; 7 vs 14 days	Microplastic release increased from 7 to 14 days. PET-based aligners released the most MPs, while Graphy released the least.
Choi et al. (2025) [28]	Korea/USA	In vitro mechanical/viscoelastic study	Temperature-responsive 3D-printed resin; TC-85	n = 3/temperature group; different thicknesses	Higher temperature reduced strength and modulus but improved stress relaxation and shape-memory recovery.
Khalil and Zaher (2025) [29]	Egypt	In vitro comparative study	3D-printed shape-memory resin; Tera Harz TC-85 DAC	96 specimens; printing orientations and thicknesses compared	Printing orientation did not affect flexural strength, while 0.7 mm specimens showed higher strength than 0.5 mm specimens.

Table 5 presents the material composition, manufacturing method, thickness, reported properties, advantages, and limitations of the tested aligner materials. PETG-based materials were frequently reported for transparency, formability, and stiffness, whereas TPU and PU-based materials were mainly associated with elasticity, hardness, and potential comfort advantages. Direct 3D-printed resins offered manufacturing advantages but showed variable mechanical stability, roughness, leaching, and abrasion behavior. The table also demonstrates that several studies lacked complete reporting of thickness, material composition, or optical properties, limiting direct comparison across materials [7-29] (Table 5).

**Table 5:** Material types and reported properties 3D, three-dimensional; BPA, bisphenol A; BPS, bisphenol S; DMA, dynamic mechanical analysis; DPA, direct printed aligner; FTIR, Fourier-transform infrared spectroscopy; GPa, gigapascal; HPDL, human periodontal ligament; MP, microplastic; NP, nanoplastic; NR, not reported; PCT, polycyclohexylenedimethylene terephthalate; PET, polyethylene terephthalate; PETG/PET-G, polyethylene terephthalate glycol; PMMA, polymethyl methacrylate; PP/PE, polypropylene/polyethylene; PP-EPR, polypropylene-ethylene-propylene rubber; PU, polyurethane; SMP, shape-memory polymer; TFA, thermoformed aligner; TPU, thermoplastic polyurethane

Study	Material/Brand	Method/Thickness	Reported properties	Main advantage	Main Limitation
Kwok et al. (2022) [8]	SmartTrack, Zendura, Essix C+, Essix Ace, Tru-Tain DX30-25; PCT/TPU, TPU, PP-EPR, PETG/PCT	Commercial sheets or preformed aligners; 0.74-1.00 mm where reported	Transparent/amorphous materials except PP-EPR; flexural modulus 0.9-1.7 GPa	Zendura and Essix Ace showed high stiffness; multilayer SmartTrack combined PCT core and TPU skin	Some compositions/additives were not fully identified; no aging data
Xiang et al. (2021) [9]	Conventional PETG and modified PETG	Fabricated clear aligners; 0.75 mm	Modified PETG showed higher elasticity and abrasion resistance	Modified PETG had better force stability and less surface damage	The exact modified formulation was not reported
Divakar et al. (2026) [10]	Erkodur PETG, Taglus PU, Zendura PU	Thermoformed over 3D-printed models; 0.75-0.80 mm	Roughness, hardness, and flexural strength were assessed	PETG showed the highest flexural strength after aging; PU showed higher hardness	Intraoral aging increased PETG roughness and reduced PU flexural strength
Dalaie et al. (2021) [11]	Duran and Erkodur PETG	Pressure thermoformed; 0.8-1.0 mm	Elastic/storage modulus and thermal properties reported	Duran showed better thermal and mechanical stability	Only PETG materials tested; no occlusal fatigue loading
Cui et al. (2025) [12]	Biolon, Duran, Erkodur, Essix ACE, Essix C+; PETG, copolyester, PP/PE	Commercial sheets; 0.89-1.02 mm	Tensile modulus ranged from 1.06 to 2.07 GPa	Erkodur had the highest modulus; Essix ACE showed stable stress behavior	High temperature caused rapid stress relaxation
Elshazly et al. (2023) [13]	Essix ACE copolyester/PET-based material	Thermoformed aligners; 0.75 mm	Force and torque generation measured	Stable force/torque behavior in water and artificial saliva	Only one material tested; no continuous loading model
Fang et al. (2013) [14]	Erkodur, Biolon, Masel, Keystone, Duran; PETG, PET, copolyester	Commercial sheets; 1.0 mm	Stress relaxation measured	Erkodur and Masel showed slower relaxation	Materials were not thermoformed; a short 3-hour test only
Bhate and Nagesh (2024) [15]	Duran PET-G and Zendura PU	Thermoformed samples; 0.75 mm	Surface roughness and flexural strength assessed	Duran maintained stable roughness after aging	Flexural strength decreased in both materials; Zendura roughness increased
Das et al. (2026) [16]	Invisalign, Smile, Illusion aligners; PU-based, PETG-based, unknown material	Commercial aligners sectioned for extraction; NR	Cell viability and cytotoxicity were assessed	Invisalign showed the highest biocompatibility	Illusion showed dose-dependent cytotoxicity; exact composition unclear
Pendse et al. (2025) [17]	TAC, Flash, Taglus Premium, Invisalign SmartTrack; PET-G, PET, multilayer PU	Prefabricated aligners ground for testing; NR	MTT absorbance/cell viability reported	No significant cytotoxicity observed	Cell viability percentage and exposure details were limited
Zecca et al. (2025) [18]	Graphy TC-85-DAC and Invisalign SmartTrack	Direct printed vs thermoformed; 0.5-0.57 mm	MP/NP release and surface roughness assessed	TFA released fewer and smaller particles than DPA	DPA released larger and more numerous particles
Puchert et al. (2026) [19]	Dental LT Clear V2, V Print Splint Comfort, TC-85 DAC, Biolon	3D printed vs thermoformed PET-G; test-dependent thickness	Flexural modulus, hardness, creep, abrasion, and water uptake were assessed	Biolon PET-G remained stable after water storage	3D-printed materials lost modulus/hardness; some cracked
Šimunović et al. (2023) [20]	Duran+, CA Pro, Zendura A, Zendura FLX; PETG, TPU, multilayer materials	Vacuum thermoformed; 0.75-0.76 mm	Flexural modulus, roughness, and FTIR were assessed	Multilayer and TPU materials showed aging stability	Duran+ showed a significant modulus change after 1000 thermocycles
Chen et al. (2025) [21]	LC, GY, RD, ED, Biolon SC; 3D-printed and thermoformed materials	Direct printed or thermoformed; 0.75 mm	Light transmittance, roughness, color stability, and biofilm were assessed	Graphy GY performed best among the 3D-printed groups	LC and RD showed more roughness, discoloration, or bacterial adhesion
Cintora-López et al. (2023) [22]	Polyurethane aligner material	Commercial aligners; 0.72 mm	Creep, elongation, and water absorption were assessed	Useful constant deformation behavior under orthodontic loads	Thermocycling shortened the effective deformation phase
Chen et al. (2023) [23]	Duran T, Biolon, Zendura FLX; PETG, PET, TPU/PU	Commercial films; 0.75 mm	Strength decay after saliva/beverage immersion was assessed	Zendura FLX showed better resistance to beverage-related strength loss	PET/PETG showed greater strength changes in some beverages
Daniele et al. (2020) [24]	Erkodur, Essix Plastic, Ghost Aligner, Zendura; PETG/PET and PU	Commercial disks; thickness NR	Elastic modulus, tensile strength, transparency, color, and water absorption were assessed.	Zendura had the highest modulus and tensile strength	Zendura had lower transparency and higher water absorption
Lo et al. (2024) [25]	Duran, Essix, Leone, Maxflex, Zendura, Keystone; PETG, TPU, PET	Original and thermoformed sheets; NR	HPDL cell viability assessed	Most materials maintained >70% cell viability	Thermoformed PETG showed reduced viability after 14-day immersion
Bandić et al. (2025) [7]	Zendura A, Zendura FLX, Erkoloc-pro, CA Pro+; TPU and multilayer materials	Non-thermoformed and thermoformed; 0.75-1.0 mm	Oral fibroblast viability assessed	Most materials remained above acceptable viability thresholds	Cytotoxicity occurred in selected high-concentration/long-exposure groups
Wendl et al. (2026) [26]	Graphy TC-85 DAC, SMP-Aligner, CA Pro, Erkodur-al	Direct printed, SMP, and thermoformed aligners; NR	Leachables, BPA, and BPS assessed	Conventional thermoformed materials released fewer compounds	Graphy released more leachables; BPS was detected only in Graphy
Orilisi et al. (2026) [27]	Alleo, F22, FlexiLigner, Graphy, Invisalign, KeySplint, Lineo, LuxCreo, Spark, SureSmile	Thermoformed and direct-printed aligners; NR	MP number, size, morphology, and polymer type assessed	Graphy showed the lowest MP release	PET-based aligners released the highest MP levels
Choi et al. (2025) [28]	TC-85 temperature-responsive 3D-printed resin	Direct 3D printing; 0.5-0.8 mm	Modulus, stress relaxation, creep, and shape recovery were assessed	Temperature-responsive recovery may allow gentler force delivery	Water sorption and long-term oral simulation were limited
Khalil and Zaher (2025) [29]	Tera Harz TC-85 DAC shape-memory resin	Direct 3D printing; 0.5 and 0.7 mm	Flexural strength assessed by orientation and thickness	Direct printing avoids thermoforming; 0.7 mm showed higher strength	Flat specimens used; no saliva aging or mastication simulation

Table 6 outlines the main experimental methods used to assess clear aligner materials, including DMA, tensile testing, three-point bending, stress relaxation, force/torque simulation, thermocycling, cytotoxicity assays, chemical leaching analysis, and microplastic testing. The wide variation in test methods, aging media, loading protocols, temperatures, and outcome units indicates substantial methodological heterogeneity. This heterogeneity is clinically important because differences in artificial saliva exposure, thermocycling, intraoral wear, and chewing simulation may influence force retention, modulus, roughness, biocompatibility, and degradation outcomes [7-29] (Table 6).

**Table 6:** Testing characteristics of the included studies AFM, atomic force microscopy; ATR-FTIR, attenuated total reflection Fourier-transform infrared spectroscopy; BPA, bisphenol A; BPS, bisphenol S; CCK-8, Cell Counting Kit-8; DMA, dynamic mechanical analysis; DMTA, dynamic mechanical thermal analysis; DSC, differential scanning calorimetry; E′, storage modulus; E″, loss modulus; GC-MS, gas chromatography–mass spectrometry; GC-MS/MS, gas chromatography–tandem mass spectrometry; GPa, gigapascal; HPDL, human periodontal ligament; HS-SPME, headspace solid-phase microextraction; HV, Vickers hardness; MP, microplastic; MPa, megapascal; N, newton; NBS, National Bureau of Standards color unit; Nmm, newton-millimeter; OD, optical density; OM, optical microscopy; OMSS, Orthodontic Measurement and Simulation System; PETG/PET-G, polyethylene terephthalate glycol; PP/PE, polypropylene/polyethylene; PU, polyurethane; Ra, average surface roughness; SEM, scanning electron microscopy; SMP, shape-memory polymer; T0, baseline/as received; T1, post-thermoforming; T2, post-aging or post-intraoral use; TEM, transmission electron microscopy; Tg, glass transition temperature; TPU, thermoplastic polyurethane; UV-Vis, ultraviolet-visible spectroscopy; VHN, Vickers hardness number

Study	Material(s)	Test Method	Aging/Experimental Condition	Main Outcome/Unit
Kwok et al. (2022) [8]	SmartTrack, Zendura, Essix C+, Essix Ace, Tru-Tain DX30-25	DMA, dual cantilever	No aging; room temperature testing	Flexural modulus, GPa/MPa
Xiang et al. (2021) [9]	Conventional and modified PETG	Thin-film pressure sensor	Artificial saliva at 37°C for 0-14 days	Force decay/stability, N
Divakar et al. (2026) [10]	Erkodur PETG, Taglus PU, Zendura PU	Profilometry, Vickers hardness, three-point bending	T0, T1, T2 after 2-week intraoral aging	Roughness, hardness, flexural strength; μm, VHN, MPa
Dalaie et al. (2021) [11]	Duran and Erkodur PETG	Three-point bending, Vickers hardness, DSC, DMTA	Thermoforming and simulated 2-week thermocycling	Flexural modulus, hardness, Tg, E′/E″; MPa, HV, °C
Cui et al. (2025) [12]	PETG, copolyester, PP/PE	Tensile and stress relaxation tests	Temperature exposure and 2-week wet immersion	Tensile modulus, strength, residual stress; GPa, MPa, %
Elshazly et al. (2023) [13]	Essix ACE	OMSS force/torque simulation	Deionized water or artificial saliva at 37°C for 14 days	Force and torque decay; N, Nmm
Fang et al. (2013) [14]	Erkodur, Biolon, Masel, Keystone, Duran	Dynamic stress relaxation	Ambient air or 37°C water bath for 3 h	Initial/residual stress; MPa, %
Bhate and Nagesh (2024) [15]	Duran PET-G, Zendura PU	Surface profilometry and three-point bending	T0, T1, T2 after 2-week simulated aging	Surface roughness and flexural force; μm, N
Das et al. (2026) [16]	Invisalign, Smile, and Illusion aligners	MTT cytotoxicity assay	30-day saline extraction; 48 h cell exposure	Cell viability/cytotoxicity; %
Pendse et al. (2025) [17]	TAC, Flash, Taglus Premium, Invisalign SmartTrack	MTT cytotoxicity assay	Cell exposure at T1, T2, and T3	Cell viability/cytotoxicity; absorbance/%
Zecca et al. (2025) [18]	Graphy TC-85-DAC, Invisalign SmartTrack	Friction test, OM, TEM, AFM, gravimetry	120 rubbing cycles and sonication at 37°C	Plastic residue, particle coverage/size, roughness; g, %, µm², nm
Puchert et al. (2026) [19]	Biolon, Dental LT Clear V2, V Print Splint Comfort, TC-85 DAC	Three-point bending, indentation, chewing simulation, and hygroscopic test	14-day water storage and 10,000 chewing cycles	Modulus, hardness, creep, abrasion, water uptake; MPa, N/mm², mm³, %
Šimunović et al. (2023) [20]	Duran+, CA Pro, Zendura A, Zendura FLX	Three-point bending, profilometry, ATR-FTIR	500 and 1000 thermocycles	Flexural modulus, roughness, chemical composition; GPa, Ra, cm⁻¹
Chen et al. (2025) [21]	LC, GY, RD, ED, Biolon SC	Profilometry, haze meter, colorimetry, biofilm assay	Saliva, tea, coffee, and S. mutans exposure	Roughness, transmittance, color change, bacterial adhesion; Ra, %, ΔE00, OD490
Cintora-López et al. (2023) [22]	Polyurethane aligner	Creep/deformation test	Artificial saliva, 0-720 h, thermocycling, 0-30 N loading	Deformation, creep, water absorption; mm, N, %
Chen et al. (2023) [23]	Duran T, Biolon, Zendura FLX	Three-point bending	Saliva and beverages for 1, 4, and 7 days	Initial force and residual strength ratio; %
Daniele et al. (2020) [24]	Erkodur, Essix Plastic, Ghost Aligner, Zendura	Tensile test, DMA, UV-VIS, colorimetry, SEM, water absorption	Staining for 7-14 days; water absorption up to ~350 h	Stress, strain, modulus, color/transparency, water absorption; MPa, %, NBS, absorbance, μg/mm³
Lo et al. (2024) [25]	Duran, Essix, Leone, Maxflex, Zendura, Keystone	MTT assay and optical microscopy	7- and 14-day extracts; cells assessed on days 1, 3, and 5	HPDL cell viability/cytotoxicity; %
Bandić et al. (2025) [7]	Zendura A, Zendura FLX, Erkoloc-pro, CA Pro+	CCK-8 cytotoxicity assay	14-day artificial saliva storage; 4-72 h cell exposure	Cell metabolic activity/viability; % of control
Wendl et al. (2026) [26]	Graphy TC-85 DAC, SMP-Aligner, CA Pro, Erkodur-al	HS-SPME-GC-MS and GC-MS/MS	Artificial saliva and methanol extraction for 24-72 h	Leachables, BPA, BPS; μg/g
Orilisi et al. (2026) [27]	Ten thermoformed and 3D-printed aligner brands	Friction protocol, ATR-FTIR, Raman, SEM	Simulated 7- and 14-day wear/friction	MP number, size, morphology, polymer type; count, μm
Choi et al. (2025) [28]	TC-85 resin	Tensile test, DMA, stress relaxation/creep, shape-memory test	Water bath or temperature-controlled testing at 30-45°C	Strength, modulus, force, creep, recovery; MPa, N, %
Khalil and Zaher (2025) [29]	Tera Harz TC-85 DAC	Three-point bending	Immediate testing at 37°C; different thicknesses/orientations	Flexural strength; MPa

Table 7 summarizes the cytotoxicity and cell viability findings across the included studies. Overall, most clear aligner materials showed acceptable biocompatibility, with cell viability generally remaining above the commonly used 70% safety threshold. Invisalign and Smile Aligners showed high viability in HGF cells, while Illusion Aligners showed a dose-dependent reduction with slight cytotoxicity [16]. Pendse et al. reported no statistically significant cytotoxicity among PET-G, PET, and PU-based materials, although percentage viability was not provided [17]. Lo et al. found that most PETG, TPU, and PET materials remained biocompatible, but thermoformed PETG showed reduced viability after longer extraction [25]. Bandić et al. showed that cytotoxicity was mainly concentration- and time-dependent, with selected materials falling below 70% only under prolonged or high-concentration exposure, while CA Pro+ showed the most favorable profile [7] (Table 7).

**Table 7:** Cytotoxicity and cell viability findings of the included studies C3, highest eluate concentration; DMEM, Dulbecco's Modified Eagle Medium; HGF, human gingival fibroblasts; HOF, human oral fibroblasts; HGMSC, human gingival mesenchymal stem cells; HPDL, human periodontal ligament cells; ISO, International Organization for Standardization; NR, not reported; OD, optical density; PET, polyethylene terephthalate; PETG/PET-G, polyethylene terephthalate glycol; PU, polyurethane; T1-T3, study-defined time points; TPU, thermoplastic polyurethane

Study	Material(s)/Cell Line	Extraction/Exposure	Cell Viability/Cytotoxicity Level	Main Conclusion
Das et al. (2026) [16]	Invisalign; HGF	Normal saline extract diluted to 5%, 10%, and 20%; 48 h exposure	97.0%-98.2%; no cytotoxicity	Invisalign showed the highest biocompatibility with a negligible dose-related decline.
Das et al. (2026) [16]	Smile Aligners; HGF	Normal saline extract diluted to 5%, 10%, and 20%; 48 h exposure	94.1%-99.6%; no cytotoxicity	Smile Aligners showed acceptable cytocompatibility across all tested concentrations.
Das et al. (2026) [16]	Illusion Aligners; HGF	Normal saline extract diluted to 5%, 10%, and 20%; 48 h exposure	79.7%-91.5%; slight cytotoxicity at 10% and 20%	Illusion Aligners showed the greatest dose-dependent reduction in cell viability.
Pendse et al. (2025) [17]	TAC PET-G, Flash PET, Taglus Premium PET, Invisalign SmartTrack PU; HGMSC/HGF-type cells	DMEM extract/particle exposure for 24 h; T1-T3 reported	Viability percentage NR; OD values reported; no significant cytotoxicity	All tested materials showed no statistically significant cytotoxicity, although percentage viability was not reported.
Lo et al. (2024) [25]	Duran, Essix, Leone PETG; HPDL cells	DMEM extracts from 7- and 14-day immersion; cells assessed on days 1, 3, and 5	Exact values NR; mostly >70%, but thermoformed PETG showed lower viability after 14-day extraction.	Thermoforming and longer extraction may reduce PETG biocompatibility, although most values remained within acceptable limits.
Lo et al. (2024) [25]	Zendura and Maxflex TPU; HPDL cells	Same as above	Reported >70%; acceptable biocompatibility	TPU materials maintained cell viability above the safety benchmark.
Lo et al. (2024) [25]	Keystone PET; HPDL cells	Same as above	Reported >70%; acceptable biocompatibility	PET maintained cell viability above the accepted threshold.
Bandić et al. (2025) [7]	Zendura A; HOF	Artificial saliva eluate diluted in culture medium; 4, 24, 48, and 72 h exposure.	Exact values NR; c3 mean <70% after 72 h	Cytotoxicity occurred at the highest eluate concentration after prolonged exposure.
Bandić et al. (2025) [7]	Zendura FLX; HOF	Same as above	Mostly >70%; isolated non-thermoformed c3 minimum <70% after 72 h	Generally acceptable cytotoxicity profile, especially after thermoforming.
Bandić et al. (2025) [7]	Erkoloc-pro; HOF	Same as above	Exact values NR; selected values <70%	Cytotoxicity appeared concentration- and time-dependent.
Bandić et al. (2025) [7]	CA Pro+; HOF	Same as above	All values >70%; non-cytotoxic	CA Pro+ showed the most favorable cytotoxicity profile among the tested materials.

Table 8 summarizes evidence related to BPA detection and chemical leaching. Most studies did not directly quantify BPA or leachable compounds. Kwok et al. characterized polymer composition and additives but did not perform a BPA release assay [8], while Xiang et al. and Daniele et al. evaluated surface chemistry or material degradation without direct BPA quantification [9,24]. Wendl et al. provided the most relevant chemical-leaching data, showing that BPA was not detected in the tested aligners, while BPS was detected only in Graphy TC-85 DAC. Graphy and SMP-Aligner released more leachable compounds than conventional thermoformed materials, whereas CA Pro and Erkodur-al showed low or absent leaching profiles [26]. Studies not included in Table 8 because they did not assess BPA or chemical release were Bandić et al., Divakar et al., Dalaie et al., Cui et al., Elshazly et al., Fang et al., Bhate et al., Nagesh et al., Das et al., Pendse et al., Zecca et al., Puchert et al., Šimunović et al., Chen et al., Cintora-López et al., Chen et al., Lo et al., Orilisi et al., Choi et al., and Khalil and Zaher [7,10-23,25,27-29] (Table 8).

**Table 8:** BPA and chemical leaching findings of the included studies ATR-FTIR, attenuated total reflection Fourier-transform infrared spectroscopy; BPA, bisphenol A; BHT, butylated hydroxytoluene; BPS, bisphenol S; DSC, differential scanning calorimetry; FTIR-ATR, Fourier-transform infrared spectroscopy with attenuated total reflection; GC-MS/MS, gas chromatography–tandem mass spectrometry; HS-SPME-GC-MS, headspace solid-phase microextraction gas chromatography–mass spectrometry; NMR, nuclear magnetic resonance; PETG, polyethylene terephthalate glycol; PU, polyurethane; SMP, shape-memory polymer; TGA, thermogravimetric analysis; UV-Vis, ultraviolet-visible spectroscopy

Study	Material(s)	Medium/Method	Exposure Duration	BPA Finding	Other Chemicals Detected	Main Conclusion
Kwok et al. (2022) [8]	SmartTrack, Zendura, Essix C+, Essix Ace, Tru-Tain DX30-25	NMR, ATR-FTIR, TGA, DSC; solvent dissolution testing	Overnight dissolution	Not assessed	Polymer composition and some unidentified additives were reported	Useful for material composition, but not applicable to BPA/leaching analysis.
Xiang et al. (2021) [9]	Conventional and modified PETG	Artificial saliva aging: ATR-FTIR surface analysis	0 and 14 days	Not assessed	Surface chemical groups identified	Surface chemistry was evaluated, but BPA or leachable compounds were not quantified.
Daniele et al. (2020) [24]	Erkodur, Essix Plastic, Ghost Aligner, Zendura	FTIR-ATR, UV-VIS, water absorption/solubility tests	7–14 days staining; up to ~350 h water absorption	Not assessed	Material loss/solubility discussed, especially for PU at high temperatures	Physical and chemical degradation were assessed, but BPA release was not measured.
Wendl et al. (2026) [26]	Graphy / Tera Harz TC-85 DAC	Artificial saliva and methanol extraction; HS-SPME-GC-MS and GC-MS/MS	24, 48, 72 h; bisphenol testing after 72 h	Not detected; <0.01 μg/g	BPS 0.1 μg/g; isoborneol, benzaldehyde derivative, isobornyl acetate, and 1,3-dioxane derivative detected	Graph showed the highest chemical release; most leaching occurred within the first 24 h.
Wendl et al. (2026) [26]	SMP-Aligner	Same as above	Same as above	Not detected; <0.01 μg/g	Propylene carbonate, 2-butoxyethyl acetate, and a possible adipate/dioxacyclododecane compound were detected.	SMP-Aligner released fewer compounds than Graphy but more than conventional thermoformed aligners.
Wendl et al. (2026) [26]	CA Pro Clear Aligner	Same as above	Same as above	Not detected; <0.01 μg/g	BHT was detected only in volatile screening; no saliva leachables were detected	CA Pro showed a very low leaching profile.
Wendl et al. (2026) [26]	Erkodur-al	Same as above	Same as above	Not detected; <0.01 μg/g	No leachable compounds detected in artificial saliva	Erkodur-al showed the most chemically stable profile among the tested materials.

Table 9 summarizes studies assessing microplastic release, surface roughness, abrasion, or surface degradation. Surface deterioration was commonly observed after saliva aging, intraoral wear, thermocycling, or simulated friction. Xiang et al. showed greater surface damage in conventional PETG than modified PETG [9], while Divakar et al. reported the greatest roughness increase in Erkodur PETG after intraoral wear [10]. Bhate and Nagesh found stable roughness in Duran but increased roughness in Zendura after aging [15]. Zecca et al. and Orilisi et al. directly assessed particle release and showed that simulated friction can generate microplastics, with direct-printed or PET-based aligners showing greater particle release in some comparisons [18,27]. Studies not included in Table 9 because they did not assess microplastic release, roughness, abrasion, or surface degradation were Bandić et al., Kwok et al., Dalaie et al., Cui et al., Elshazly et al., Fang et al., Das et al., Pendse et al., Lo et al., Wendl et al., Choi et al., and Khalil and Zaher [7,8,11-14,16,17,25,26,28,29] (Table 9).

**Table 9:** Microplastic release and surface-degradation findings AFM, atomic force microscopy; DPA, direct printed aligner; ED, easyDu; GY, Graphy Tera Harz Clear TC85; LC, iLuxclear/Dental Clear Aligner; LT, Dental LT Clear V2; MP, microplastic; PET, polyethylene terephthalate; PETG/PET-G, polyethylene terephthalate glycol; PU, polyurethane; RD, RightBio/Right Appliance; SC, Biolon; SEM, scanning electron microscopy; T1, simulated 7-day wear or first time point; T2, simulated 14-day wear or second time point; TC-85, Tera Harz TC-85 DAC; TFA, thermoformed aligner; VP, V-Print Splint Comfort

Study	Material(s)	Wear/Loading Simulation	Duration	MP Detected	Surface/Particle Finding	Main Conclusion
Xiang et al. (2021) [9]	Conventional and modified PETG	Artificial saliva immersion; no chewing/wear simulation	14 days	Not assessed	SEM showed rougher surface, peeling, and pores in conventional PETG; modified PETG showed less damage	Modified PETG showed better surface stability after saliva aging.
Divakar et al. (2026) [10]	Erkodur PETG, Taglus PU, Zendura PU	Real intraoral wear	2 weeks	Not assessed	Roughness increased most in Erkodur PETG: 0.040 ± 0.003 to 0.500 ± 0.016 μm; Taglus: 0.139 to 0.160 μm; Zendura: 0.033 to 0.118 μm	PETG showed the greatest surface roughness increase after intraoral aging.
Bhate and Nagesh (2024) [15]	Duran PET-G, Zendura PU	Thermoforming and simulated aging; no brushing/wear simulation	Simulated 2 weeks	Not assessed	Duran roughness remained stable: 0.07 to 0.06 μm; Zendura increased from 0.06 to 0.25 μm	Zendura showed a significant roughness increase, while Duran remained stable.
Zecca et al. (2025) [18]	Graphy TC-85-DAC DPA and Invisalign SmartTrack TFA	120 rubbing cycles under 1 kg force, followed by sonication	Short-term friction simulation	Yes	DPA showed larger plastic particles and higher particle coverage; TFA released fewer/smaller particles but had higher AFM roughness	Direct-printed aligners released larger and more numerous particles than thermoformed aligners.
Puchert et al. (2026) [19]	Biolon PET-G, Dental LT Clear V2, V Print Splint Comfort, TC-85 DAC	Chewing simulation with 10,000 cycles, 50 N, and thermocycling	10,000 cycles	Not directly measured	Abrasion volume: Biolon 1.12 ± 0.37 mm³; LT 0.72 ± 0.21 mm³; VP 1.05 ± 0.23 mm³; TC-85 2.01 ± 0.40 mm³	LT showed better abrasion resistance; TC-85 and VP showed cracking in several specimens.
Šimunović et al. (2023) [20]	Duran+, CA Pro, Zendura A, Zendura FLX	Thermocycling only; no mechanical wear/loading	500 and 1000 cycles	Not assessed	Roughness changes were small and statistically non-significant across materials.	Thermocycling did not significantly alter surface roughness.
Chen et al. (2025) [21]	LC, GY, RD, ED, Biolon SC	Artificial saliva immersion	45 days	Not assessed	Roughness increased in all groups; LC showed the highest irregularities, while thermoformed ED and SC remained smoother.	Thermoformed materials showed smoother surfaces than most 3D-printed groups.
Cintora-López et al. (2023) [22]	Polyurethane aligner	Constant loading with thermocycling in artificial saliva	Up to 720 h / 1 month	Not assessed	Surface roughness not assessed	The study focused on creep, deformation, and water absorption rather than MP release.
Chen et al. (2023) [23]	Duran T, Biolon, Zendura FLX	Immersion in saliva and beverages	1, 4, and 7 days	Not assessed	Surface roughness not assessed	The study evaluated force/strength decay, not MP release or roughness.
Daniele et al. (2020) [24]	Erkodur, Essix Plastic, Ghost Aligner, Zendura	Water/staining immersion	14 days staining; water absorption up to ~350 h	Not assessed	SEM showed surface irregularities, ripples, deformation, or swelling-related changes after immersion	Immersion and temperature exposure altered surface morphology, especially in PU.
Orilisi et al. (2026) [27]	Alleo, F22, FlexiLigner, Graphy, Invisalign, KeySplint, Lineo, LuxCreo, Spark, SureSmile	Mechanical friction simulation	7 and 14 days	Yes	MP release increased from T1 to T2; PET-based aligners showed the highest MP release; Graphy showed the lowest MP release; Spark showed reduced mean MP size from 38.95 ± 10.47 μm to 14.36 ± 7.68 μm	Longer simulated wear increased MP release across aligner systems.

Table 10 presents studies that reported outcomes indirectly relevant to clinical performance, including force stability, stress relaxation, simulated tooth movement forces, flexural force, creep, deformation, abrasion, and shape-memory behavior. Direct clinical outcomes, such as tooth movement accuracy, retention force, and patient-reported comfort, were not reported in the included studies. Xiang et al. showed that modified PETG maintained a more stable orthodontic force than conventional PETG [9]. Elshazly et al. provided simulated tooth movement data, reporting force and torque values for intrusion, extrusion, translation, and rotation [13]. Cintora-López et al. and Choi et al. contributed clinically relevant information on deformation behavior and temperature-responsive force delivery, respectively, but without direct clinical validation [22,28]. Studies not included in Table 10 because they did not assess clinical-performance-related outcomes were Bandić et al., Kwok et al., Dalaie et al., Fang et al., Das et al., Pendse et al., Zecca et al., Šimunović et al., Chen et al., Chen et al., Daniele et al., Lo et al., Wendl et al., Orilisi et al., and Khalil and Zaher [7,8,11,14,16-18,20,21,23-27,29] (Table 10).

**Table 10:** Clinical relevance, simulated tooth movement, retention, and comfort outcomes 3D, three-dimensional; MP, microplastic; N, newton; Nmm, newton-millimeter; NR, not reported; PETG/PET-G, polyethylene terephthalate glycol; PU, polyurethane; TC-85, Tera Harz TC-85 DAC; TFA, thermoformed aligner

Study	Material(s)	Study/Wear Protocol	Clinical-Performance Outcome	Accuracy/Retention/Comfort	Main Finding
Xiang et al. (2021) [9]	Conventional and modified PETG	Artificial saliva aging for 0, 3, 7, 10, and 14 days; simulated 0.0, 0.1, and 0.2 mm activations	Orthodontic force stability	Accuracy NR; retention NR; comfort NR	Force decreased with aging and increased with higher activation; modified PETG showed higher and more stable force than conventional PETG.
Divakar et al. (2026) [10]	Erkodur PETG, Taglus PU, Zendura PU	Prospective 2-week intraoral wear with laboratory testing	Roughness, hardness, and flexural strength after clinical aging	Accuracy NR; retention NR; comfort not directly measured	PETG showed increased roughness but comparatively higher flexural strength after aging; PU materials showed higher hardness but reduced flexural strength.
Cui et al. (2025) [12]	Biolon, Duran, Erkodur, Essix ACE, Essix C+	Temperature and wet-environment simulation	Stress relaxation and residual stress	Accuracy NR; retention NR; comfort NR	Elevated temperature reduced force stability; Erkodur and Essix ACE showed relatively stable stress behavior.
Elshazly et al. (2023) [13]	Essix ACE	14-day aging in water or artificial saliva; simulated 0.2 mm intrusion/extrusion, 0.2 mm translation, and 2° rotation	Force and torque generation	Accuracy NR; retention NR; comfort NR	Vertical forces ranged 1.4-4.6 N, horizontal forces 1.3-2.5 N, and rotation torque 5.4-41.7 Nmm; aging medium had no major effect.
Bhate and Nagesh (2024) [15]	Duran PET-G and Zendura PU	Simulated 2-week aging after thermoforming	Surface roughness and flexural force	Accuracy NR; retention NR; comfort NR	Duran showed stable roughness but reduced flexural strength; Zendura showed increased roughness and reduced flexural strength after aging.
Puchert et al. (2026) [19]	Biolon, Dental LT Clear V2, V Print Splint Comfort, TC-85 DAC	14-day water storage and chewing simulation	Modulus, hardness, creep, abrasion, and water uptake	Accuracy NR; retention NR; comfort not directly measured	Dental LT approached Biolon in flexural modulus after water storage; some 3D-printed materials showed cracking during chewing simulation.
Cintora-López et al. (2023) [22]	Polyurethane aligner	Artificial saliva, thermocycling, and 0, 3, and 30 N loading for up to 720 h	Creep and deformation behavior relevant to force maintenance	Accuracy NR; retention NR; comfort NR	A constant deformation plateau may support orthodontic correction, but thermocycling and higher load reduced this plateau.
Choi et al. (2025) [28]	TC-85 resin	Temperature-controlled testing at 30-45°C with deformation and recovery testing	Viscoelastic and shape-memory behavior relevant to force delivery	Accuracy NR; retention NR; comfort not directly measured	TC-85 may reduce excessive initial force and improve insertion/removal through temperature-responsive flexibility, but patient comfort was not clinically tested.

**Figure 2:**
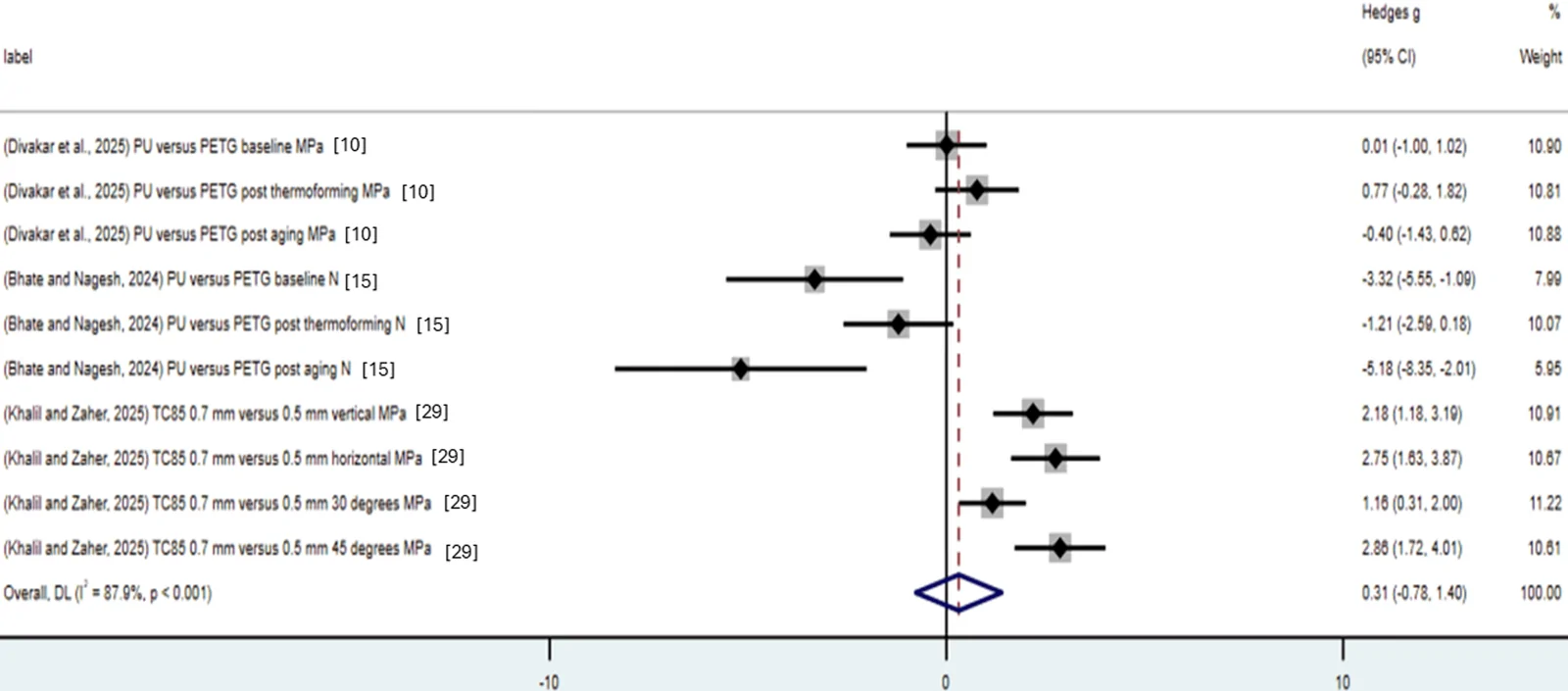
Flexural performance forest plot Source: Refs [10,15,29]

Figure 2 presents the exploratory meta-analysis of comparative flexural performance. The summary standardized mean difference was Hedges' g = 0.31 (95% CI: -0.78 to 1.40), providing no clear evidence of overall superiority for either comparison group. Heterogeneity was high (I² = 87.9%), reflecting differences in material composition, comparator selection, specimen thickness, manufacturing method, and test protocol. The estimate should therefore be interpreted as an average of heterogeneous laboratory comparisons rather than a common material effect [10,15,29] (Figure 2).

**Figure 3:**
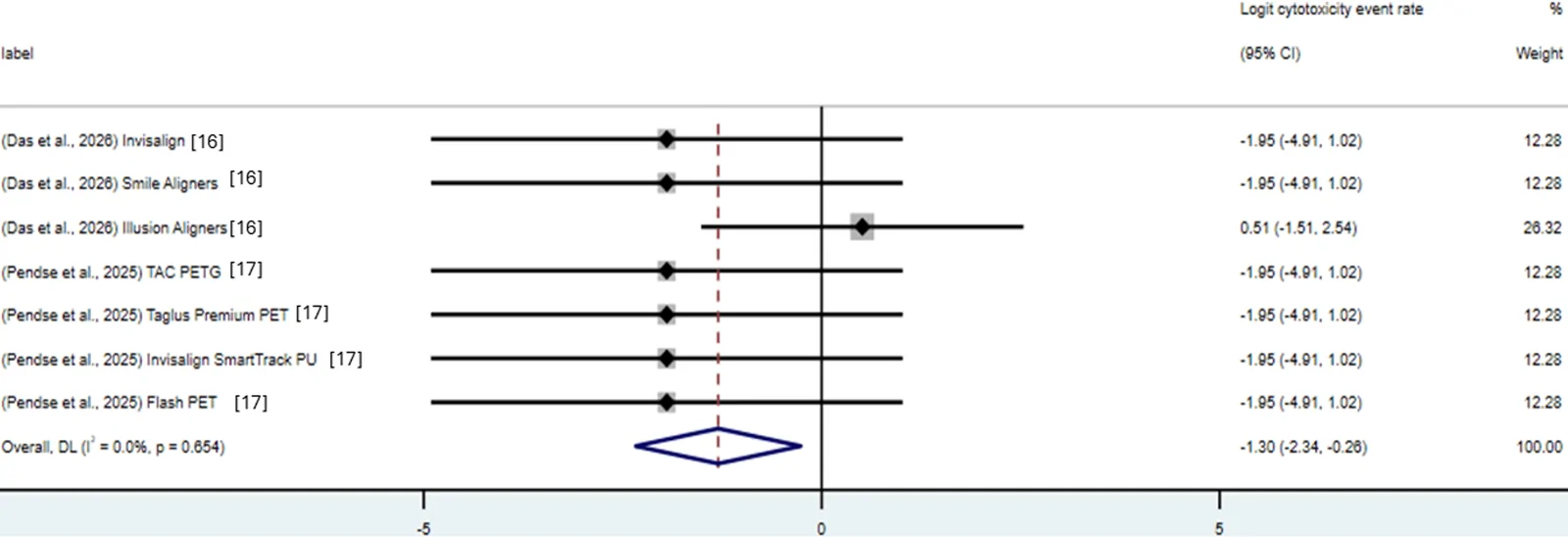
Cytotoxicity event rate forest plot Source: Refs [16,17]

Figure 3 presents an exploratory synthesis of laboratory cytotoxicity classifications. A cytotoxicity event was defined as a material or experimental condition classified by the original study as cytotoxic or demonstrating cell viability below the prespecified 70% threshold. The pooled logit estimate was -1.30 (95% CI: -2.34 to -0.26), corresponding to an estimated proportion of 21.4% (95% CI: 8.8%-43.5%). Although I² was 0%, only two independent studies contributed, and their assays differed in cell type, extraction procedure, concentration, and exposure duration. This estimate therefore does not represent a patient-level incidence or clinical probability of harm [16,17] (Figure 3).

**Figure 4:**
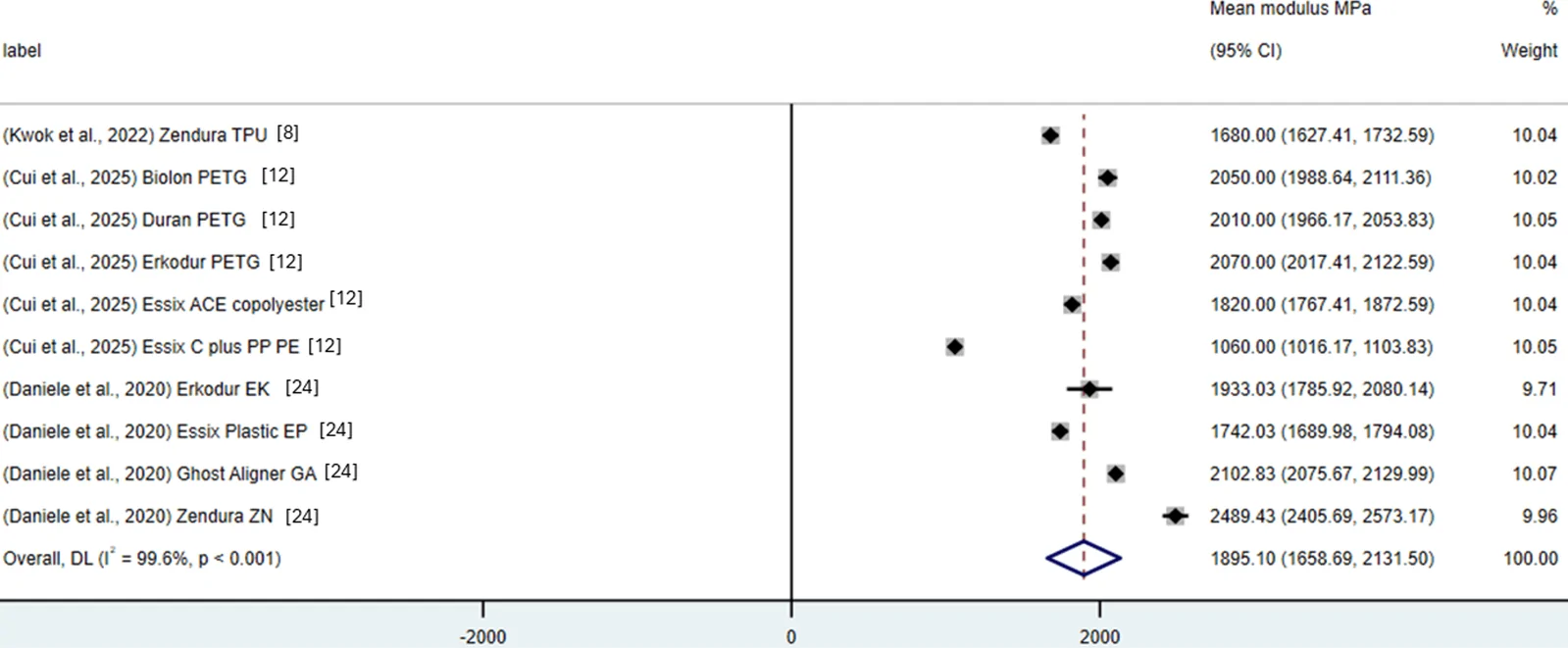
Baseline modulus forest plot Source: Refs [8,12,24]

Figure 4 summarizes baseline modulus measurements across the included material groups. The descriptive random-effects mean was 1,895.10 MPa (95% CI: 1,658.69-2,131.50 MPa). Heterogeneity was extreme (I² = 99.6%), reflecting differences in polymer composition, specimen preparation, test method, temperature, and loading configuration. The summary value should not be interpreted as an optimal or clinically recommended modulus; it represents an average across highly dissimilar laboratory measurements [8,12,24] (Figure 4).

**Figure 5:**
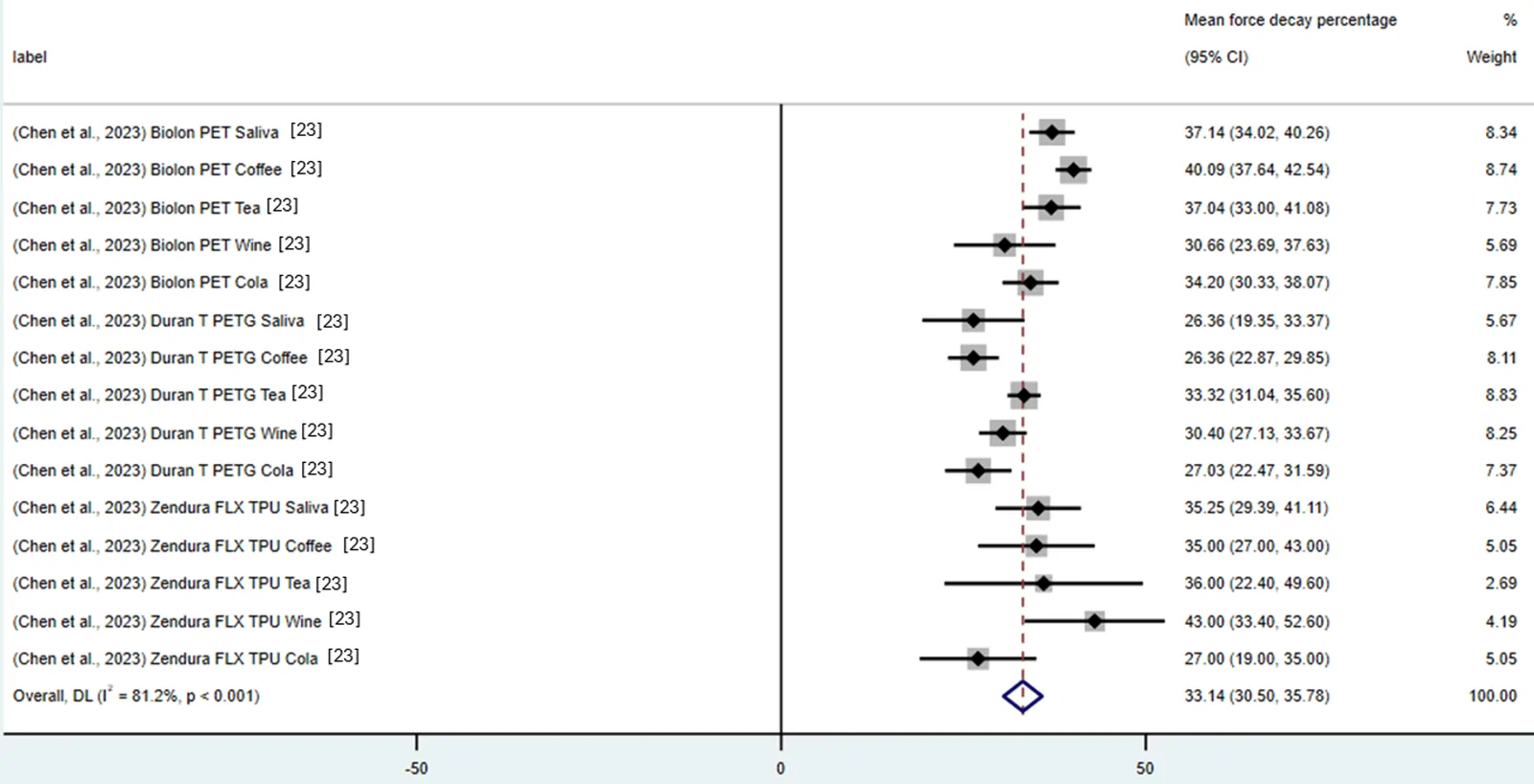
Force decay after seven days forest plot Source: Ref [23]

Figure 5 presents a within-study quantitative summary of seven-day force decay across the material and immersion conditions reported by Chen et al. [23]. The average force-decay estimate was 33.14% (95% CI: 30.50%-35.78%). Variation among the material and immersion conditions was high (I² = 81.2%). Because all observations originated from one source study and may be correlated, this result should not be interpreted as a meta-analytic estimate applicable to all clear aligner materials (Figure 5).

**Figure 6:**
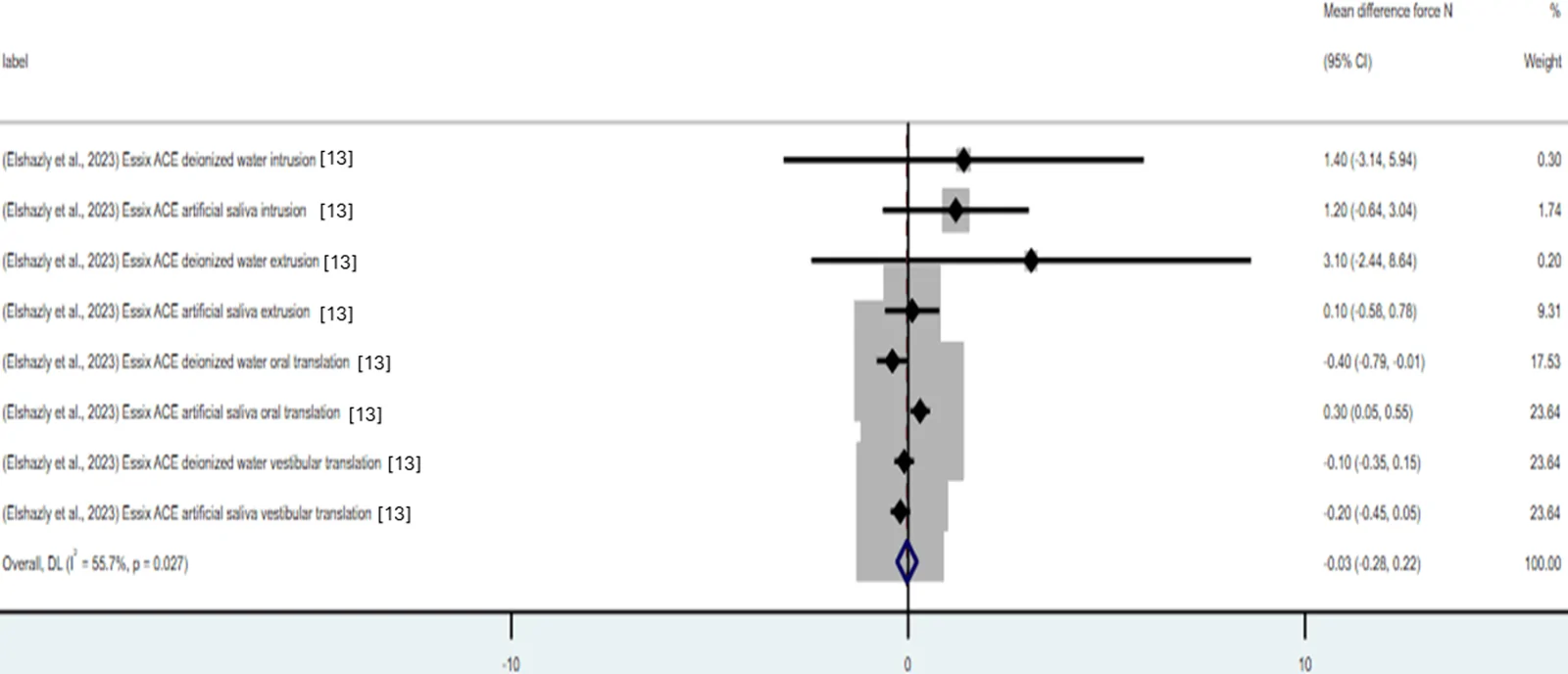
Force change after aging forest plot Source: Ref [13]

Figure 6 presents a within-study quantitative summary of force change after aging across the simulated movement conditions evaluated by Elshazly et al. [13]. The summary mean change was -0.03 N (95% CI: -0.28 to 0.22 N). Variation occurred among intrusion, extrusion, oral translation, and vestibular translation conditions. Because all observations were derived from one material and one source study, the result does not demonstrate that aging has no effect across different clear aligner materials (Figure 6).

**Figure 7:**
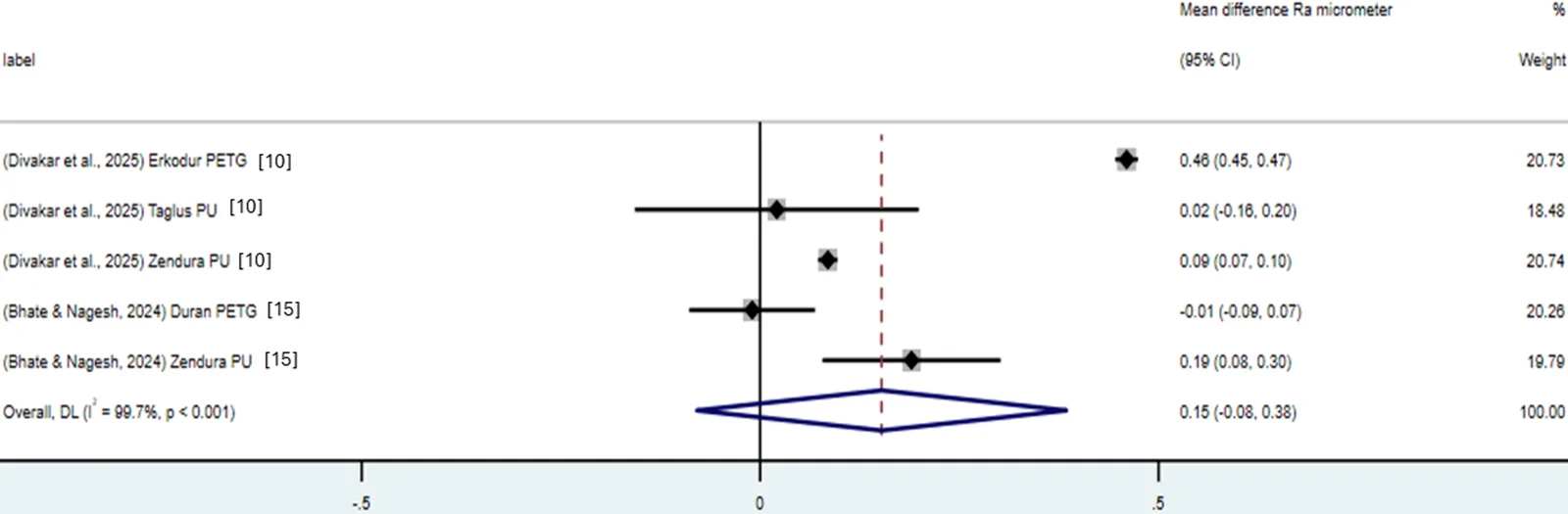
Surface roughness change forest plot Source: Refs [10,15]

Figure 7 summarizes surface-roughness change after aging or intraoral wear. The exploratory pooled mean change was 0.15 μm (95% CI: -0.08 to 0.38 μm), but heterogeneity was extreme (I² = 99.7%). The overall estimate was not statistically significant and masked substantial material- and protocol-specific variation. Erkodur PETG showed the largest increase among the included comparisons, whereas Duran PETG showed minimal change [10,15] (Figure 7).

**Figure 8:**
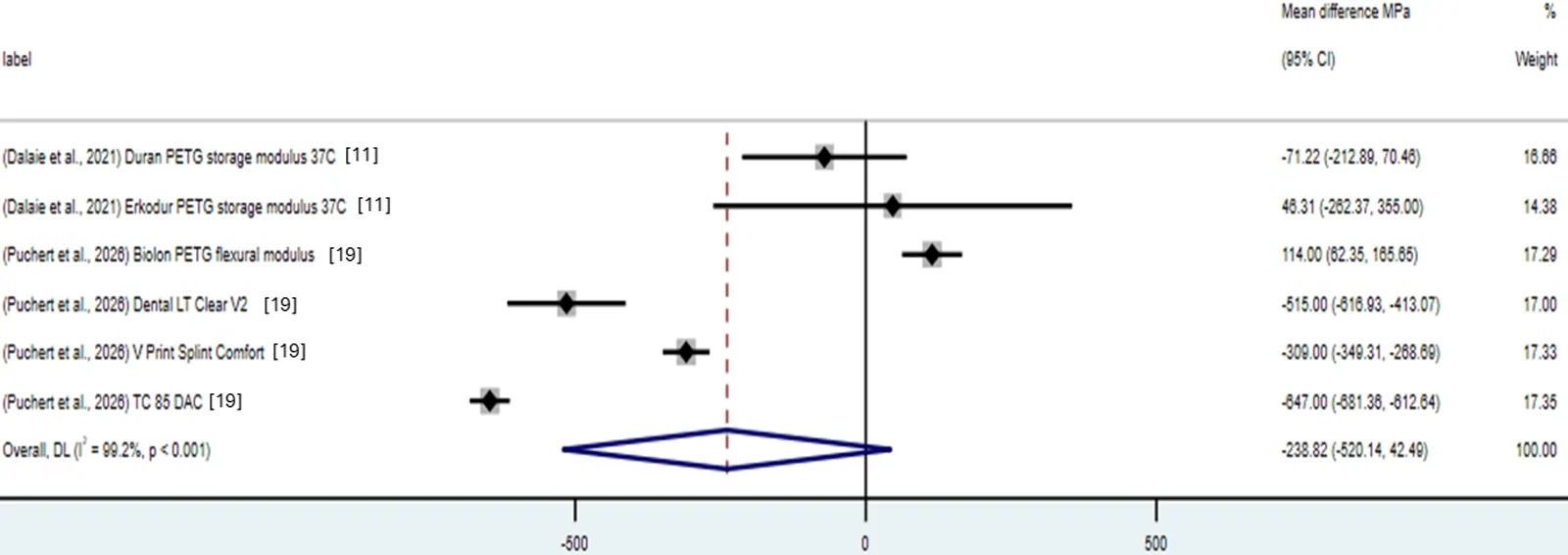
Modulus change after the aging forest plot Source: Refs [11,19]

Figure 8 presents the exploratory synthesis of modulus change after aging. The pooled mean change was -238.82 MPa (95% CI: -520.14 to 42.49 MPa), and the confidence interval included no change. Heterogeneity was extreme (I² = 98.2%), with different directions and magnitudes of change across material groups. The summary estimate should therefore be interpreted descriptively and does not establish a uniform reduction in modulus following aging (Figure 8).

**Figure 9:**
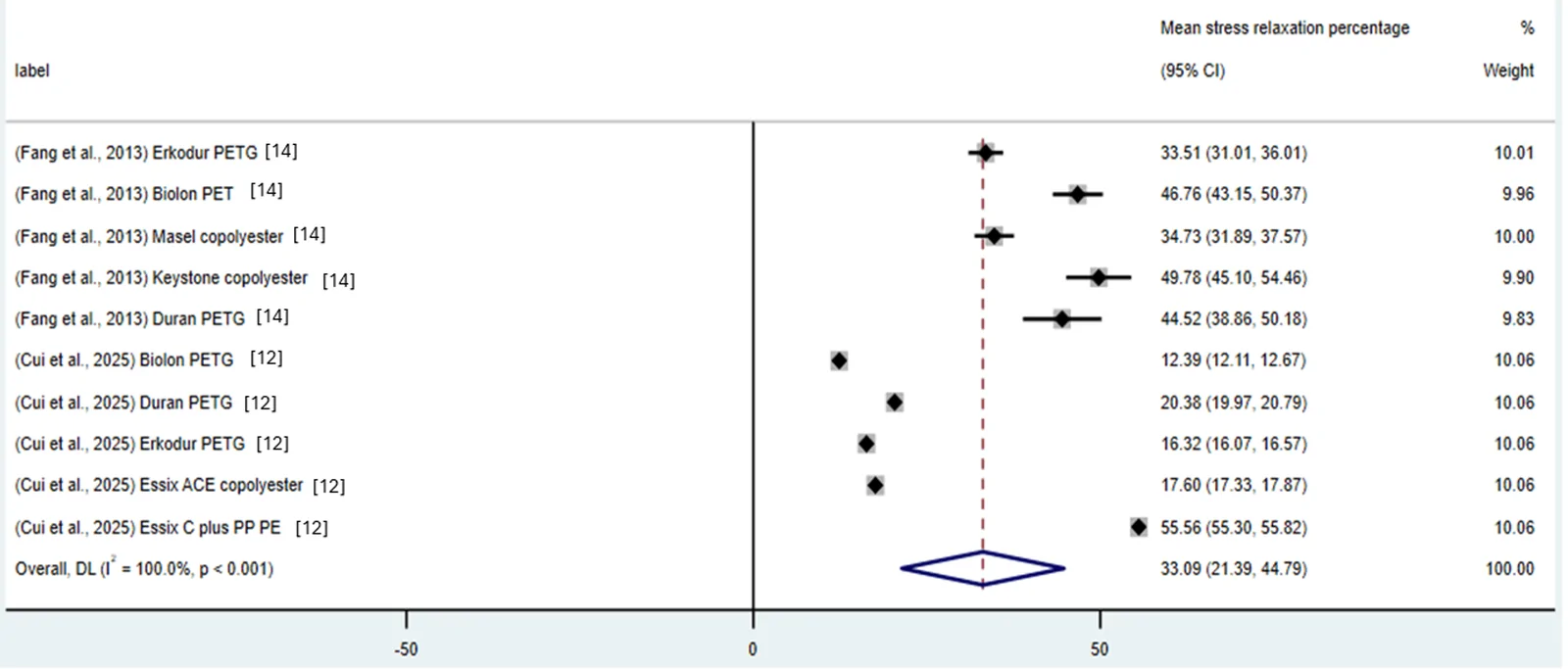
Stress relaxation percentage forest plot Source: Refs [12,14]

Figure 9 presents the exploratory synthesis of stress-relaxation percentages. The descriptive random-effects mean was 33.09% (95% CI: 21.39%-44.79%), but heterogeneity was extreme (I² = 100%). The contributing observations differed in material composition, test duration, applied strain, temperature, hydration, and environmental conditions. The pooled value therefore does not represent a common stress-relaxation rate for all clear aligner materials and should be interpreted alongside the individual study estimates [12,14] (Figure 9).

Discussion

This systematic review indicates that clear aligner materials differ in mechanical behavior, force retention, surface stability, biological response, and chemical-release profile. However, the evidence base consisted predominantly of in vitro investigations using heterogeneous materials, specimens, and testing protocols. The QUIN assessment identified no study at low risk of bias, with most studies classified as medium risk and four as high risk. The exploratory quantitative syntheses demonstrated substantial variability but did not identify a universally superior material. Extreme heterogeneity for modulus, surface roughness, modulus change, and stress relaxation limits the clinical generalizability of the corresponding summary estimates. The findings should therefore be interpreted primarily as evidence of material- and protocol-specific behavior rather than definitive comparative clinical rankings.

The mechanical findings of this review are consistent with previous experimental and review literature showing that thermoforming and aging can alter clear aligner properties. In the present meta-analysis, pooled flexural performance showed a small and non-significant overall effect, but heterogeneity was high, suggesting that the response was strongly material- and protocol-dependent. This agrees with Dalaie et al., who reported that thermoforming significantly reduced flexural modulus in PETG materials, with Duran and Erkodur showing reductions after fabrication [11]. Similarly, Bhate and Nagesh found that thermoforming and simulated aging reduced flexural strength in both Duran PETG and Zendura PU materials [15]. In contrast, some studies included in the present review showed smaller or non-significant force changes after aging, which may be explained by differences in specimen geometry, aging medium, thermocycling protocol, and testing method. Therefore, while the direction of evidence supports mechanical degradation after processing and wear, the magnitude of that degradation cannot be generalized across all materials.

PETG remains one of the most widely used clear aligner materials because of its transparency, formability, and availability. The present review found that PETG materials generally provide acceptable mechanical performance, but they may show force reduction after thermoforming, aging, or immersion in artificial saliva. This finding is supported by Dalaie et al., who showed that thermoforming had a stronger effect than aging on the thermomechanical properties of PETG aligners [11]. Bhate and Nagesh also observed reduced flexural strength in Duran PETG after thermoforming and aging [15]. However, PETG did not always perform poorly; some PETG materials retained better stability than other polymers, depending on the brand and testing method. This contrast suggests that PETG performance is not determined only by polymer category but also by formulation, sheet thickness, thermoforming temperature, cooling, and exposure conditions.

TPU and PU-based materials showed a different pattern. These materials are generally more elastic and may offer better comfort, insertion, and adaptation to dental morphology. The present results suggest that TPU and multilayer PU systems may be useful where flexibility and stress dissipation are important. This is consistent with Delgado et al., who described TPU as a material with favorable elastic behavior and better organoleptic comfort than some polyesters [30]. Multilayer systems may further balance stiffness and flexibility by combining hard and soft layers, which may reduce excessive initial force while maintaining clinically useful retention. However, the present review also found that PU materials may be affected by aging, as Bhate and Nagesh reported increased surface roughness in Zendura after simulated aging and reduced flexural strength over time [15]. Therefore, TPU and PU materials may improve comfort, but their long-term surface and mechanical stability still require careful evaluation.

Force decay and stress relaxation were consistently identified as time-dependent material behaviors, but their magnitude varied according to polymer composition, temperature, hydration, loading configuration, and test duration. The seven-day force-decay estimate originated from multiple material and immersion conditions within a single source study and should therefore be interpreted as a within-study quantitative summary rather than a general meta-analytic estimate. Fang et al. observed faster stress relaxation in a 37°C water environment than in ambient conditions [14], while Cui et al. demonstrated substantial variation according to material and temperature [12]. The stress-relaxation synthesis also showed extreme heterogeneity. Collectively, these findings support the occurrence of time-dependent force reduction but do not demonstrate that every aligner material loses approximately one-third of its force within seven days.

Surface roughness and modulus changes after aging also showed high variability. In the present review, the pooled surface roughness change after aging was small and not statistically significant, but the heterogeneity was very high. This means that some materials showed meaningful surface deterioration while others remained stable. Bhate and Nagesh found that Zendura PU showed a significant roughness increase after aging, whereas Duran PETG did not show a comparable change [15]. Similarly, studies evaluating intraoral wear and simulated aging reported that surface changes may depend on polymer type, cleaning habits, beverages, and mechanical loading [10,23]. Clinically, this is important because surface roughness may influence plaque accumulation, staining, biofilm adhesion, patient comfort, and esthetic acceptability.

Stiffness, modulus, thickness, fit, and viscoelastic behavior should be interpreted as interacting material characteristics rather than isolated predictors of clinical performance. The descriptive baseline modulus estimate showed extreme heterogeneity across PETG, TPU, PP/PE, PU, and direct-printed materials, reflecting differences in composition, specimen preparation, and testing methods. Although thickness can influence force magnitude, its effect depends on material stiffness, geometry, stress relaxation, and aligner adaptation. Therefore, neither modulus nor thickness alone can establish clinical superiority or reliably predict tooth movement without corresponding biomechanical and clinical validation [30].

Direct 3D-printed resins represent an important development in orthodontic manufacturing. They may reduce the need for physical models, decrease material waste, improve digital workflow efficiency, and allow more controlled geometry. This agrees with Bandić et al., who described direct 3D printing as a method that may reduce production time, waste, and cost [31]. Dantagnan et al. also emphasized that 3D printing could improve accuracy and reduce the environmental burden of thermoforming [32]. However, the present review found that 3D-printed aligner resins remain limited by insufficient long-term clinical data. Their performance depends heavily on printer type, resin composition, washing protocol, post-curing time, post-curing atmosphere, print orientation, and aging conditions. Thus, although direct 3D printing is promising, it cannot yet be considered clinically superior to thermoformed materials across all outcomes.

The biological safety findings were generally favorable under the laboratory conditions examined, although the quantitative cytotoxicity estimate requires cautious interpretation. Only two independent studies contributed to the event-rate analysis, and their cell lines, extraction methods, concentrations, exposure durations, and definitions of cytotoxicity differed. Consequently, an I² value of 0% should not be interpreted as evidence that the findings were universally consistent. Selected materials demonstrated reduced viability under concentrated or prolonged extraction conditions, while most tested materials remained above the commonly applied 70% cell-viability threshold. These findings suggest that cytotoxic responses depend on the tested material, processing method, extraction severity, concentration, and exposure duration rather than representing a uniform property of clear aligner materials [31,33].

The current results are also consistent with systematic reviews focused on biological safety. Dantagnan et al. reported that direct printed aligner evidence is mostly in vitro and that slight cytotoxicity may occur in some studies, particularly when compared with thermoformed aligners [32]. Delgado et al. also described most aligner materials as having low or acceptable cytotoxicity but highlighted concerns related to leachates, monomers, and microplastics [30]. The present review adds to this literature by combining conventional thermoplastics and emerging 3D-printed materials, showing that the overall cytotoxicity signal is low but not absent. Clinically, this means that currently used materials are likely acceptable when manufactured properly, but newer resins require stronger evidence from standardized and clinical studies.

Post-curing emerged as a critical factor for 3D-printed aligner safety and performance. Bouchema et al. showed that post-curing time affected mechanical properties, leachable release, and cytotoxicity, with insufficiently post-cured materials showing worse biological behavior [33]. Dantagnan et al. also reported that different curing protocols may alter cytotoxicity outcomes and that manufacturer-recommended post-processing should be followed [32]. This contrasts with conventional thermoformed materials, where the major concern is often thermoforming-related mechanical change rather than incomplete polymerization. For printed resins, incomplete curing can leave unreacted monomers, photoinitiators, and degradation products that may leach into saliva. Therefore, 3D-printed aligners should be evaluated not only by resin brand but also by a validated workflow.

BPA and chemical release remain uncertain. In the present review, BPA evidence was inconsistent and could not be interpreted as a single pooled estimate due to methodological differences. This agrees with BPA-focused evidence showing that only a small number of studies detected BPA, while many reported undetectable or very low release [33,34]. Some earlier clinical evidence reported higher salivary BPA after appliance placement, whereas several in vitro studies showed no detectable BPA from commonly used aligner materials. This inconsistency may be due to different extraction media, detection limits, follow-up times, surface-area-to-volume ratios, and material formulations. Therefore, BPA should not be dismissed, but the available evidence does not consistently show high BPA release from most clear aligner materials.

For 3D-printed resins, the more relevant chemical concern may be monomer and photoinitiator release rather than BPA alone. Dantagnan et al. reported urethane dimethacrylate (UDMA) release from direct-printed aligners, while BPA was not detected in some included studies [32]. Bouchema et al. also found that aging and post-curing influenced leachable release from printed resins [33]. This supports the interpretation that chemical safety assessment should include UDMA, 2-hydroxyethyl methacrylate (HEMA), ethylene glycol dimethacrylate (EGDMA), photoinitiators, degradation products, and bisphenol analogues rather than focusing only on BPA. The present review, therefore, expands the safety discussion from a narrow BPA-centered approach to a broader chemical-release framework.

Microplastic release is an emerging concern. The present review identified microplastic release as an important but insufficiently standardizedoutcome. Studies such as Zecca et al. and Orilisi et al. suggest that aligners can release particles during simulated wear, friction, or mechanical loading [18,27]. However, the long-term systemic implications remain unclear. Delgado et al. and Dantagnan et al. also noted growing concern about microplastics and particle release from orthodontic polymers [30,32]. In contrast to cytotoxicity and mechanical testing, microplastic research remains less mature, with limited agreement on particle-size thresholds, analytical methods, polymer identification, and biological relevance. Therefore, microplastic release should be considered a plausible exposure pathway, but current evidence is not sufficient to determine clinical harm.

The findings may inform hypotheses concerning material selection, but direct clinical evidence remains limited. The included laboratory studies did not establish that higher-stiffness materials produce more accurate bodily movement, torque, rotation, intrusion, or extrusion, and patient-reported comfort and retention were rarely examined. Clinicians should therefore avoid selecting materials solely based on isolated laboratory modulus, force, or stress-relaxation values. Material characteristics may be considered together with movement type, treatment stage, aligner geometry, fit, attachment design, oral conditions, and patient adherence; however, material-specific clinical recommendations remain provisional.

The available evidence does not justify recommending a specific 7-day, 10-day, or 14-day replacement interval solely based on laboratory force-decay findings. The within-study seven-day summary suggests that wear duration may interact with material composition, immersion environment, and movement complexity, but it does not establish an optimal clinical replacement schedule. Biological tooth movement, aligner tracking, attachment design, movement complexity, and patient adherence must also be considered. Prospective clinical comparisons are required before material-specific wear intervals can be recommended. The within-study force-decay summary suggests that wear duration may interact with material composition, immersion environment, and movement complexity, but it does not establish an optimal clinical replacement schedule. Prospective clinical studies are required before material-specific wear intervals can be recommended. The present finding of substantial seven-day force decay suggests that some materials may lose a clinically important amount of force before the end of a traditional 14-day wear interval. However, changing aligners too rapidly may also risk incomplete biological tooth movement. Therefore, 7-day, 10-day, and 14-day protocols should be evaluated separately by material type and movement complexity. Dalaie et al. and Bhate and Nagesh both used simulated two-week aging models, but the present review indicates that clinically relevant changes may occur earlier and vary between materials [11,15]. Future protocols should therefore avoid assuming that all aligners remain mechanically active for the same duration.

Patient handling and cleaning practices may also influence material behavior. Exposure to excessive heat, abrasive cleaning, or chemicals not recommended by the manufacturer may contribute to deformation, discoloration, surface changes, or material degradation. Clinicians should therefore reinforce manufacturer-specific guidance regarding aligner cleaning, storage, insertion, and removal. However, because the included evidence did not directly compare long-term clinical cleaning protocols, the relative effects of individual cleaning products or patient-care practices remain uncertain.

This review integrates mechanical, biological, chemical, optical, surface-degradation, and clinically relevant laboratory outcomes across conventional thermoplastic and emerging direct 3D-printed aligner materials. It includes PETG, TPU, PU, multilayer systems, PP/PE-based materials, copolyesters, and direct-printed resins, providing a broader evaluation than reviews limited to a single material group or outcome domain. Study-level material characteristics, testing conditions, and outcome data are presented in detailed tables to maintain transparency. An exploratory quantitative synthesis was used where compatible numerical data were available, while design-appropriate methodological appraisal was performed using QUIN and the JBI quasi-experimental checklist.

This review has several important limitations. First, 22 of the 23 included studies were predominantly in vitro investigations, and laboratory conditions cannot fully reproduce salivary flow, fluctuating oral temperature and pH, mastication, parafunctional activity, biofilm formation, repeated insertion and removal, cleaning behavior, patient adherence, or biological tooth movement. Second, the studies differed substantially in material formulation, specimen geometry, thickness, manufacturing procedures, aging media, temperature, loading, exposure duration, extraction concentration, cell type, and outcome measurement. This heterogeneity limited the interpretability of the summary estimates, particularly for modulus, surface roughness, modulus change, and stress relaxation. Third, the QUIN assessment classified most in-vitro studies as medium risk of bias and four as high risk; no study met the low-risk threshold. Common deficiencies included absent sample-size calculations, incomplete sampling descriptions, limited randomization and blinding, inadequate operator or assessor reporting, and incomplete statistical details. Because QUIN judgments depend on published reporting, some studies may have implemented safeguards that were not described in their articles. The prospective clinical/laboratory study was assessed separately and remained limited by its small sample, absence of random allocation, and limited control of patient-level intraoral exposures.

Fourth, several quantitative syntheses contained multiple material groups or experimental conditions from the same source study. These observations may be correlated, and analyses based on one source study should be interpreted as within-study quantitative summaries rather than independent meta-analyses. The small number of independent studies contributing to each outcome also limited reliable estimation of heterogeneity and prevented meaningful meta-regression, sensitivity analysis, and publication-bias testing. An I² value of 0% based on only a few studies should not be interpreted as proof of consistency. Fifth, some studies did not report complete means, standard deviations, sample sizes, material compositions, or experimental details, restricting quantitative synthesis. The English-language and full-text restrictions may also have introduced language and availability bias. In addition, the review was not prospectively registered, and no publicly accessible protocol was prepared. Readers therefore cannot compare the completed review with a time-stamped prespecified plan, and unrecorded deviations from the original review decisions cannot be completely excluded.

Recent secondary literature published between 2024 and 2026 reinforces the principal findings of this review. Reviews of biological safety indicate that the cytocompatibility of thermoplastic and resin-based aligners depends on polymer composition, extraction conditions, post-curing, aging, and exposure duration [30-40]. Reviews of mechanical performance similarly demonstrate substantial variation among PETG, TPU, PU, PP, PC, EVA, multilayer polymers, and shape-memory resins in relation to stiffness, force delivery, thickness response, stress relaxation, and aging stability [41-50]. Emerging publications have also emphasized chemical leaching, microplastic release, resin post-processing, and environmental impact [32-45,50]. These recent reviews were used to contextualize the findings, but were not included as primary studies because secondary reviews were excluded under the predefined eligibility criteria. The updated literature confirms that direct clinical comparisons remain scarce and that claims of superiority for one material or manufacturing method remain premature. Evidence on thermo-mechanical aging also shows that force transmission may change after simulated oral exposure, reinforcing the clinical relevance of aging protocols [36]. Moreover, recent literature on shape-memory resins and next-generation 3D-printed aligners suggests promising digital workflow advantages but also confirms that clinical validation remains limited [39,43,50]. Finally, environmental discussions indicate that aligner material waste and microplastic release should be considered alongside clinical performance and patient safety [44].

Future research should prioritize standardized testing and clinical translation. Mechanical studies should compare PETG, TPU, multilayer polymers, EVA, PP, PC, and 3D-printed resins under identical conditions. Clinical trials should evaluate material performance by movement type, including rotation, bodily movement, intrusion, extrusion, torque, and retention. Wear protocols of 7, 10, and 14 days should be tested separately for each material. Chemical studies should use standardized detection methods for BPA, bisphenols, monomers, photoinitiators, and degradation products. Microplastic studies should quantify particle release under realistic brushing, chewing, insertion-removal, and cleaning conditions. Manufacturers should also report material composition, curing parameters, post-processing instructions, and safety testing transparently. Long-term clinical and environmental safety studies are needed before newer direct printed aligner systems can be considered equivalent or superior to established thermoformed materials.

## Conclusions

Clear aligner materials demonstrate substantial variation in mechanical behavior, aging response, surface stability, cytocompatibility, and chemical-release profile. PETG remains widely used because of its transparency and thermoformability, while TPU, polyurethane, multilayer systems, and direct 3D-printed resins provide different combinations of stiffness, elasticity, and manufacturing flexibility. However, the available evidence does not establish the universal superiority of any material category. Most biological-safety studies reported acceptable cell viability under standard laboratory conditions, although adverse responses occurred with selected materials, concentrated extracts, prolonged exposure, or inadequate post-curing. Evidence regarding BPA was limited and inconclusive, while residual monomers, photoinitiators, other leachable compounds, and microplastic particles remain emerging concerns. The findings require cautious interpretation because the evidence was predominantly in vitro; no in vitro study was classified as having low risk of bias using QUIN, several outcomes were informed by few independent studies, some summaries incorporated multiple conditions from a single source study, and heterogeneity was frequently high or extreme. Consequently, the pooled estimates should not be translated directly into clinical material-selection or wear-schedule recommendations. Standardized testing, transparent reporting of material composition and post-processing, and prospective clinical studies linking material properties with tooth-movement accuracy, retention, comfort, adverse effects, and replacement intervals are required before firm clinical recommendations can be made.
